# Exploring the immunomodulatory role of virtual memory CD8^+^ T cells: Role of IFN gamma in tumor growth control

**DOI:** 10.3389/fimmu.2022.971001

**Published:** 2022-10-18

**Authors:** Constanza Savid-Frontera, Maria Estefania Viano, Natalia S. Baez, Nicolas L. Lidon, Quentin Fontaine, Howard A. Young, Lene Vimeux, Emmanuel Donnadieu, Maria Cecilia Rodriguez-Galan

**Affiliations:** ^1^ Inmunología CIBICI-CONICET Facultad de Ciencias Químicas, Universidad Nacional de Córdoba, Córdoba, Argentina; ^2^ Cancer Innovation Laboratory, Center for Cancer Research, National Cancer Institute, Frederick, MD, United States; ^3^ Université Paris Cité, CNRS, INSERM, Equipe Labellisée Ligue Contre le Cancer, Institut Cochin, F-75014 Paris, France

**Keywords:** virtual memory CD8^+^ T cells, cancer, IL-12, IL-18, IFNg

## Abstract

Virtual memory CD8^+^ T cells (T_VM_) have been described as cells with a memory-like phenotype but without previous antigen (Ag) exposure. T_VM_ cells have the ability to respond better to innate stimuli rather than by TCR engagement, producing large amounts of interferon gamma (IFNγ) after stimulation with interleukin (IL)-12 plus IL-18. As a result of the phenotypic similarity, T_VM_ cells have been erroneously included in the central memory T cell subset for many years. However, they can now be discriminated *via* the CD49d receptor, which is up-regulated only on conventional memory T cells (T_MEM_) and effector T cells (T_EFF_) after specific cognate Ag recognition by a TCR. In this work we show that systemic expression of IL-12 plus IL-18 induced an alteration in the normal T_VM_ vs T_MEM_/T_EFF_ distribution in secondary lymphoid organs and a preferential enrichment of T_VM_ cells in the melanoma (B16) and the pancreatic ductal adenocarcinoma (KPC) tumor models. Using our KPC bearing OT-I mouse model, we observed a significant increase in CD8^+^ T cell infiltrating the tumor islets after IL-12+IL-18 stimulation with a lower average speed when compared to those from control mice. This finding indicates a stronger interaction of T cells with tumor cells after cytokine stimulation. These results correlate with a significant reduction in tumor size in both tumor models in IL-12+IL-18-treated OT-I mice compared to control OT-I mice. Interestingly, the absence of IFNγ completely abolished the high antitumor capacity induced by IL-12+IL-18 expression, indicating an important role for these cytokines in early tumor growth control. Thus, our studies provide significant new information that indicates an important role of T_VM_ cells in the immune response against cancer.

## 1 Introduction

The ability of CD8^+^ T cells to respond to cytokines in a bystander T cell receptor (TCR)-independent way has been studied for a long time. The work of Slifka et al. examined the effects of more than 1800 cytokine combinations on virus-specific CD8^+^ T-cell activation, demonstrating that certain cytokine combinations could synergize to induce antigen-independent IFNγ production, CD69 up-regulation and in some cases exhibit differential regulatory functions (1).

Interestingly, a recent review by Maier et al. has summarized data demonstrating that recognition of cancer cells is mainly restricted to a small subset of antigen (Ag)-specific tumor infiltrating leukocytes (TILs). In fact, many TILs are “cancer ignorant” and have been defined as “bystander T cells”, that recognize non-cancer peptides, including viral antigens (2). Although the most recent evidence has pointed to bystander T cells as being the majority of infiltrating T cells in tumors, there is still a lack of consensus regarding the activation status of these cells and if their presence in tumors plays a role in anti-cancer immunity.

Within the bystander T cell subset, virtual memory T cells are a population of CD8^+^ T cells that, despite their antigen inexperience, exhibit many hallmarks compatible with conventional Ag-specific memory T cells. By consensus, the lineage markers for T_VM_ cells are: CD8^+^ CD44^hi^ CD122^hi^ CD49d^lo^. The most recent marker is integrin alpha 4 (CD49d) as it is highly expressed in T cells after a strong signal through the TCR, *via* the cognate Ag-TCR-MHC-I interaction. As a result, CD49d is highly expressed by T_MEM/_T_EFF_ but not by T_VM_ cells, thus becoming an essential marker that discriminates between these 2 populations of CD8^+^ memory T cells (3–5). Also, a transcription factor, eomesodermin (Eomes), is highly expressed by T_VM_ cells and is associated with their functional capacity (6, 7).

During their thymic maturation these “memory-like” cells can be differentiated into two populations: T_VM_ and T_IM_ (innate memory) by the expression of different markers, including CD5^hi^ in T_VM_ and interleukin (IL)-4R^hi^ and CD49d^hi^ in T_IM_ cells. However, when they reach secondary lymphoid organs (SLO), T_IM_ cells downregulate CD49d, and as such, CD5 and IL-4R expression is not sufficient to discriminate between both cell types (8). As a result, they became indistinguishable from each other and compose a heterogeneous group of memory-like cells designated as T_VM_ cells (3, 8, 9).

In spite of never having contacted their specific Ags, T_VM_ cells are capable of developing a powerful cytotoxic response in a TCR-independent manner, mainly through mechanisms that may involve interaction through the receptor NKG2D (10, 11). Also, their lytic capacity is mediated by production of large amounts of the interferon gamma (IFNg), especially after IL-12 and IL-18 stimulation (3, 12–14) and by granzymes release (15, 16). Even though these cells have been described as being important in controlling infectious diseases through innate bystander mechanisms, the role of T_VM_ cells in cancer has not been widely studied.

Interleukin-12 and IL-18 are inflammatory cytokines mainly produced by activated macrophages and dendritic cells at the initiation of an immune response (17). It has been reported that IL-12 and IL-18 are capable of activating virtual memory CD8^+^ T cells leading to rapid production of IFNγ, resulting in crucial pathogen control during certain viral and bacterial infectious processes (10, 18–21). Moreover, we have previously described the role of T_IM_ cells during a murine model of parasitic infection with *Trypanosoma cruzi (* 13).

Infiltration of CD8^+^ T cells into tumors is associated with a better prognosis for response in cancer patients (22, 23). Moreover, it has been long assumed that CD8^+^ T cells present in tumors are conventional memory CD8^+^ T specific for tumor antigens. In this context, the role of T_VM_ cells present in tumors and their Ag-independent antitumor mechanisms has not been thoroughly investigated.

The few reports that have addressed the role of T_VM_ cells (or cells compatible with T_VM_ phenotype, not identified by the current consensus lineage markers) mainly focus on the antitumor mechanism mediated by the receptor NKG2D (10, 11). However, the presence and activity of T_VM_ vs T_MEM_ cells in tumor growth control has not been thoroughly studied, especially with regard to the role of effector mechanisms. This is quite important, especially with respect to tumors that do not express the ligands for NKG2D. In this context, IFNγ is a cytokine highly produced by T_VM_ cells, able to promote a potent antitumor activity especially through anti-angiogenic mechanisms (24). Importantly, we have recently demonstrated that after IL-12 systemic expression, a significant reduction is observed in the number of blood vessels present in B16 and EL-4 tumors (25). This data is quite relevant to the biology of these tumors as they do not express NKG2D ligands (26) and their growth seems to rely upon neovascularization (24).

Based on this evidence, we decided to focus on the role of IFNγ in murine cancer models utilizing cell lines that lack NKG2D ligands, in order to avoid interference with this potential cytotoxic mechanism (26). Previously, our group demonstrated that systemic expression of IL-12+IL-18 was capable of inducing a strong antitumor effect against 2 different tumor cells lines *in vivo* (B16, melanoma and 3LL, lung carcinoma). However this occurred at the expense of toxic side effects resulting in only 50% survival over a period of 50 days post-treatment (27). Because toxicity was associated with IL-12 but not to IL-18 (27, 28), we used lower doses of IL-12 cDNA to minimize the side effects. Utilizing this lower dose, we demonstrated that survival reached 100% and the antitumor effect was still present (25). Interestingly, IL-12 systemic expression does not affect the total number of CD8^+^ T cells but induces an increase in the percentage of IFNγ^+^CD8^+^ infiltrating B16 and EL-4 tumors (25). As we have reported, a rapid and strong antitumor effect early after IL-12+IL-18 expression (27), we speculated that T_VM_ (CD8^+^ CD44^hi^ CD122^hi^ CD49d^lo^) along with tumor Ag-specific CD8^+^ effector T cells (T_EFF_) and T_MEM_ (CD8^+^ CD44^hi^ CD122^hi^ CD49d^hi^) could be playing a role in tumor growth control in an Ag-independent manner.

In the present work we have evaluated the balance between these populations in SLO and tumors, both in steady-state conditions and after systemic stimulation with IL-12+IL-18. We observed that the number and the phenotypic characteristics of these T cells experienced changes from a normal state to inflammatory T helper 1 conditions following cytokine expression. Moreover, systemic expression of IL-12+IL-18 leads to a prevalence of T_VM_ cells expressing IFNγ in tumors and defines these cells as an essential factor for tumor growth control in the early stages of disease.

## 2 Material and methods

### 2.1 Mice

Female and male WT C57BL/6, IL4KO (C57BL/6J-IL-4tm1Nnt), IFNARKO (Ifnar1tm1Ag), IFNγKO (B6.129S7-Ifngtm1Ts/J), and OT-I (RAG-sufficient, B6 background) used in this study were 6–7-weeks-old and were maintained under specific pathogen-free conditions. Animal care was provided in accordance with the procedures outlined in the Guide for the Care and Use of Laboratory Animals (NIH-Publication No. 86-23, 1985). The experimental protocols were approved by the Institutional Animal Care and Use Committee of Centro de Investigaciones en Bioquímica Clínica e Inmunología (CIBICI), Consejo Nacional de Investigaciones Científicas y Técnicas (CONICET). Our animal facility has obtained NIH animal welfare assurance (assurance no. A5802-01, Office of Laboratory Animal Welfare, NIH, Bethesda, MD, USA).

### 2.2 Cell lines

B16-F10 melanoma cells were obtained from the American Type Culture Collection (ATCC). KPC pancreatic ductal adenocarcinoma cells were previously used at Dr. Donnadieu´s labororatory (INSERM U1016, Institut Cochin, Paris, France). Both cell lines were free of Mycoplasma infection (tested by PCR every 12 months). B16-F10 melanoma cells were cultured in DMEM and KPC in DMEM/F12, both containing 10% Fetal Bovine Serum (FBS), 100 U/ml penicillin, 2mM L-Glutamine, 100 µg/ml streptomycin at 37°C, 5% CO_2_.

### 2.3 Hydrodynamic cDNA injections

The hydrodynamic gene transfer procedure was described previously by our group (13, 25, 27–30). The designated amount of each DNA was suspended in 1.6 mL of sterile 0.9% sodium chloride solution. Animals were injected in the tail vein with the cDNAs in less than 8 s and separated in two groups, control: 15 µg of ORF empty vector cDNA and IL-12+IL-18: 1 µg of IL-12 cDNA (pscIL-12, p40-p35 fusion gene) plus 10µg of IL-18 cDNA (pDEF pro-IL-18). All the expression plasmids utilize the human elongation 1-α promoter to drive transcription.

### 2.4 *In vivo* tumor models

WT, OT-I, IL4KO, IFNARKO and IFNγKO mice were shaved and injected subcutaneously (s.c.) in the left flank with 1×10^6^ B16-F10 cells or 10×10^6^ KPC cells in 100µl of a sterile 0.9% sodium chloride solution. After 7-10 days, when solid tumors were visible (4–5 mm diameter), mice were hydrodynamically injected (HI) with the designated cDNAs (Day 0). At the specified time points, tumor growth was monitored with a caliper and tumor volume was calculated as:


$$Tumorvolume:\frac{(d^{2})\times D}{2}$$

in which “*d”* corresponds to the lower diameter of the tumor, and “*D”* to the longest one. At day 7 post-hydrodynamic injection, animals were euthanized and tumors were removed and weighed using an analytical scale. 

### 2.5 Tissue processing

At day 7 post-HI, spleens, tumor-draining lymph nodes (dLN) and tumors were harvested and mechanically disrupted with a disposable mesh (FiltraBags). Cell suspensions were collected and stained for flow cytometry analysis. B16-tumors were harvested, weighed, cut into small pieces, mechanically disrupted, and resuspended at 1g of tumor/7mL of Phosphate Buffered Saline (PBS) + 10% FBS for flow cytometry analysis. KPC tumors were harvested, weighed, cut into small pieces, mechanically and enzimatically (with DNAse and Liberase)disrupted, and counted. Five million cells were stained for flow cytometry analysis. Splenocyte suspensions were depleted of red cells by treatment with ACK lysis buffer before staining.

### 2.6 Flow cytometry

Phenotypic analysis of cells from spleens, dLN and B16-F10 tumors, was performed by flow cytometry *ex vivo* on day 7 post-HI. Samples were first washed with PBS and stained with Zombie Acqua Fixable Viability Kit (Biolegend) 15 minutes at room temperature for exclusion of dead cells. Expression of different surface markers was assessed by staining with appropriate combinations of the following monoclonal antibodies (mAbs) for 30 min at 4°C: CD4 (RM4-5, Biolegend), CD8 (53-6.7, Biolegend), CD44 (IM7, eBioscience), CD45 (30-F11, Biolegend), CD49d (R1-2, Biolegend), CD122 (TM-β1, Biolegend), NK1.1 (PK136, Biolegend) and TCRb (H57-597, Biolegend) or its respective isotype matched antibody. Cells were washed twice with PBS and acquired on a BD LSR Fortessa X-20 cytometer (BD Biosciences).

To detect intranuclear Eomes expression, cells were stained for surface markers, washed, and fixed with IC Fixation Buffer (eBioscience) for 90 minutes at 4°C. Cells were washed with Permeabilization Buffer (eBioscience) and incubated for 30 minutes with the same buffer. Cells were centrifuged and incubated with the Eomes PE anti-mouse Ab (Dan11mag, eBiosciencce), or isotype-matched antibody for 45 min at 4°C and then acquired in a BD LSR Fortessa X-20.

For Ovalbumin (OVA)-specific CD8^+^ T cell detection, cells were L/D stained, washed and then stained using H-2K(b) chicken ova amino acids 257–264 SIINFEKL APC-labeled Tetramer (ProImmune or NIH Tetramer Core Facility) for 30min, followed by washing steps and surface staining. Cells were acquired in a BD LSR Fortessa X-20. Analysis was performed using FlowJo VX software (Tree Star, Inc.).

### 2.7 Tumor slice imaging

For studying CD8^+^ infiltrating T cells in KPC tumors, tumors from control and 12+18-treated OT-I mice were harvested and placed in a PLP solution (containing PFA 1%, Lysine, Sodium Periodate (NaIO4) in PBS) for 2 hours at RT, washed with PBS and embedded in 5% low-gelling-temperature agarose (type VII-A, Sigma-Aldrich) prepared in PBS.

Real-time imaging experiments were performed with KPC tumor specimens obtained 2–4 h after tumor resection from control or 12+18-treated OT-I mice at day 7 post-HI. Tumor slices were prepared as previously described (31, 32). In brief, samples were embedded in 5% low-gelling-temperature agarose (type VII-A, Sigma-Aldrich) prepared in PBS. In both cases, 200 µm slices were cut with a vibratome (VT 1000S, Leica) in a bath of ice-cold PBS. Slices were transferred to 0.4-μm organotypic culture inserts (Millicell, Millipore) in 35-mm Petri dishes containing 1 ml phenol red free RPMI 1640in an incubator at 37°C and 5% CO_2_. Live vibratome sections were stained for 15 min at 37°C with the following antibodies: PerCP-eFluor710-conjugated anti-CD8 (53-6.7, eBiosciences), BV421-conjugated anti-EpCAM (G8.8, BD Horizon) and eFluor660-conjugated anti-gp38 (8.1.1, eBioscience andwashed thereafter. All antibodies were diluted in phenol red free RPMI and used at a concentration of 10 μg/ml. To concentrate the antibodies on the tissue, a stainless-steel ring was placed in the agarose surrounding the slice.

Tumor slices were imaged with a DM500B upright microscope equipped with a SP5 confocal head (Leica) and a 37°C thermostat controlled chamber. For dynamic imaging, tumor slices were secured with a stain-less steel slice anchor (Warner Instruments) and perfused at a rate of 1 ml/min with a solution of phenol red free RPMI and then, bubbled with 95% O2 and 5% CO2. Ten minutes later, images from a first microscopic field were acquired with a 25× water immersion objective (Olympus, 20×/0.95 NA). For four-dimensional analysis of cell migration, stacks of 6–10 sections (*z* step = 5–7 μm) were acquired every 30 s for 20–40 min, at depths up to 80 μm. Regions were selected for imaging when tumor parenchyma, stroma and T cells were simultaneously present in the same microscopic field. For most of the tumors included in the study, between 2 and 4 microscopic fields were selected for time-lapse experiments.

### 2.8 Image data analysis

Image analysis was performed at the Cochin Imaging Facility (Institut Cochin, Paris). A 3D image analysis was performed on *x*, *y*, and *z* planes using Imaris 7.4 (Bitplane AG). First, superficial planes from top of the slice to 15 μm in depth were removed to exclude T cells located near the cut surface. Cellular speed means were calculated using Imaris. Tracks >10% of the total recording time were included in the analysis. To reveal the relationship between CD8^+^ T cell motility and the tumor structure (tumor islets and stroma), confocal time-lapse images of T cells were superimposed onto the corresponding gp38 and EpCAM images. CD8^+^ T cells localized in the stroma were distinguished from those infiltrated in tumor cell nests by looking at individual planes along the *z* axis. Videos and images were made by compressing the *z* information into a single image using Imaris. When a drift in the *x*, *y* dimension was noticed, it was corrected using the “Correct 3D Drift” plug-in in Image J. For the automated detection of resident CD8^+^ T cells in different tumor areas (stroma or tumor islets), we used the Image J software. First, fluorescent images threshold was obtained and converted to binary images. Angles between the cell trajectory vectors, which are the connecting lines between starting points and end points of each track, and tumor- stroma boundaries were calculated using Image J software. Only the cells positioned within a maximum distance of 100 μm from the tumor-stroma interfaces were included in further analysis.

### 2.9 Statistical analysis

Statistical analyses were performed using GraphPad Prism version 7.0 (GraphPad Software). Data was analyzed by means of a Student unpaired *t* test (comparison of 2 experimental groups), One-way analyses of variance (ANOVA) test (for comparing more than 2 experimental groups) or Two-way ANOVA test (composition of more than 2 variables). Results are expressed as means ± SEM and were considered statistically significant when p< 0.05.

## 3 Results

### 3.1 Antitumor effect of systemic IL-12+IL-18 expression

In order to induce systemic expression of the cytokines IL-12 and IL-18, we have used hydrodynamic injection as a tool. By rapidly injecting naked cDNA encoding these cytokines i.v, mostly hepatocytes uptake the cDNA and systemically and transiently produce the protein of interest. This methodology was first described by Liu et al. (33) and has been used in our laboratory in several publications (13, 25, 27–30).

Previous work from our group has shown that systemic expression of IL-12 and IL-18 is capable of inducing the expression of IL-15 in the thymus (13). When we evaluated if a similar induction was happening in the periphery, we observed that the procedure was capable of inducing the expression of IL-15 RNA in LNs, spleen and liver, the main sites of T_VM_ cell residence (34) (**Supplementary Figure 1**). Thus, we created an *in vivo* environment where 3 cytokines, important for maintenance/development and functional activities of T_VM_ cells, were simultaneously present. Moreover, the systemic amounts of IL-12 and IL-18 we induced by hydrodynamic injections were previously assessed by our laboratory and were completely tolerated by the mice and similar to what is described for certain pathological scenarios like infectious diseases (25, 27, 35–37).

**Figure 1:**
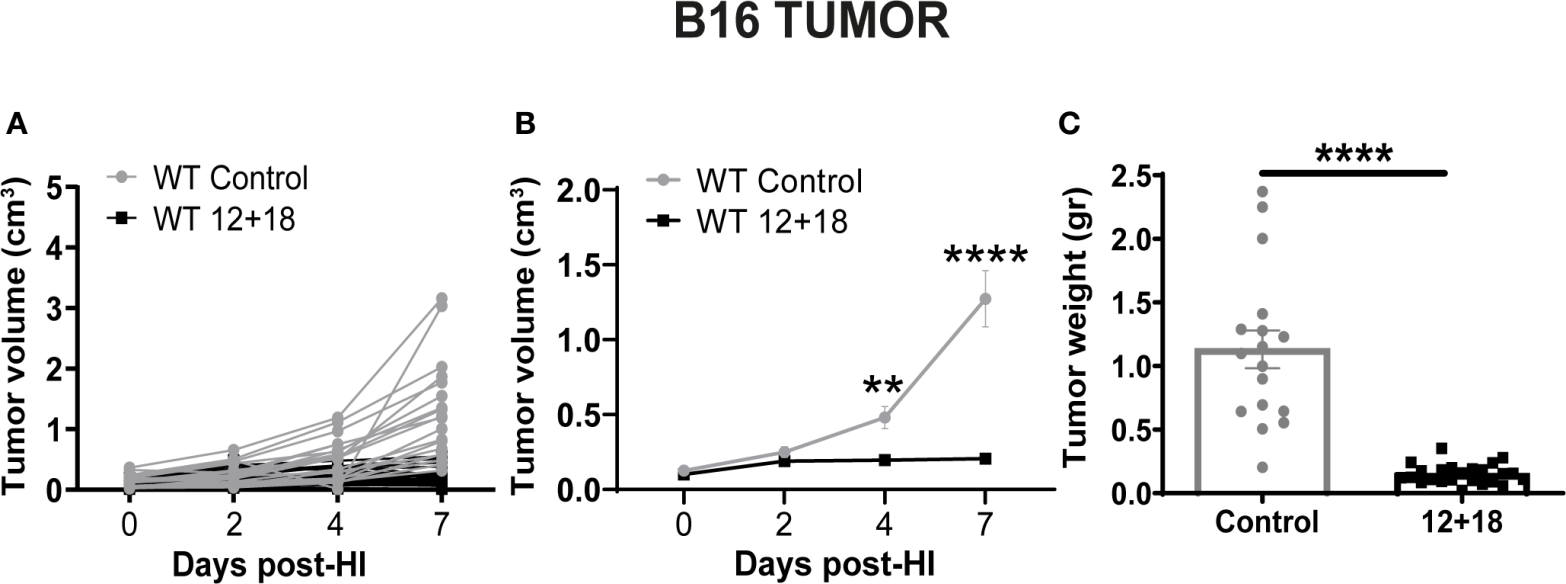
Effects of the systemic co-expression of IL-12 plus IL-18 on tumor growth. B16-bearing C57BL/6 WT mice were hydrodynamically injected (HI) with IL-12 plus IL-18 cDNA (12 + 18) or empty cDNA as the control. At the indicated time points post-HI tumor size was measured using a caliper. After 7 days post-HI mice were euthanized, and tumors were harvested and weighed using an analytical scale. **(A, B)** Tumor growth represented as tumor volume (in cm^3^) at specified days post-HI is shown **(A)** for each mouse individually or **(B)** as an average (mean ± SEM) for each group. **(C)** Tumor weight in grams is represented as mean ± SEM for each experimental group. Statistical analysis was performed using a two-way ANOVA with Sidak’s multiple comparison test on **(B)** or a Student T test in **(C)**. Values of **p<0.01, and ****p<0.0001 were consider significant.

As seen in **Figure 1**, we first determined the antitumor effect of systemic expression of IL-12 + IL-18 at the tolerated doses (see M&M section). We observed significant growth control of B16 tumors evaluated by tumor volume *in vivo* up to 7 days post-treatment (**Figures 1A, B**) as well as by tumor weight at day 7 post treatment, compared to control mice (**Figure 1C**).

### 3.2 Effects of systemic IL-12+IL-18 expression on CD8^+^ T cells in SLO and tumors

#### 3.2.1 Alterations in SLO

Taking into account that the tumor growth control was observed in a rapid time period (7 days) due to systemic cytokine stimulation, we wondered if T_VM_, T_EFF_ and early T_MEM_ cells (or pre-existing T_MEM_ cells) could be playing a role in the antitumor immune response. We focused our attention on the contribution of these cell types because they rapidly respond to IL-12, IL-18 and IL-15 stimulation as previously reported (34). While it is possible that NK cells could be also contributing, in this experimental system, NK cells are almost undetectable as we have previously reported (25, 27).

Next, we performed a comparative analysis of the frequency and phenotype of the CD8^+^ T cells in a distant (spleen) and a close (draining lymph nodes, dLNs) SLO to the tumor site in the absence (control) or presence of systemic IL-12+IL-18 (12 + 18). See strategy gates in **Supplementary Figure 2**.

**Figure 2:**
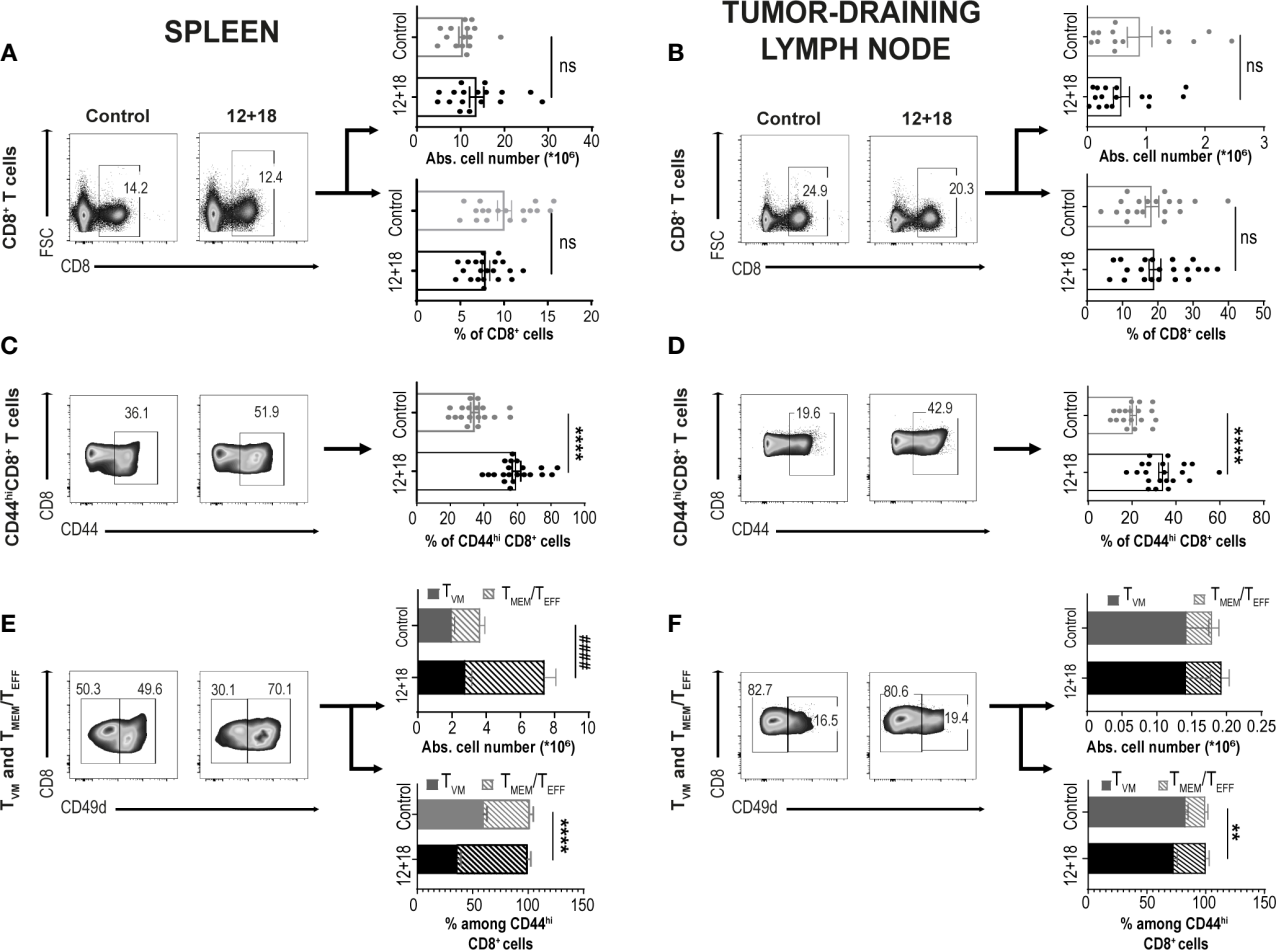
Effects of systemic co-expression of IL-12 plus IL-18 on CD8^+^ T lymphocytes in SLO. B16-bearing C57BL/6 WT mice were hydrodynamically injected (HI) with IL-12 plus IL-18 cDNA (12 + 18) or empty cDNA as the control. After 7 days post-HI mice were euthanized, and spleen and tumor-draining lymph nodes (dLN) were harvested and processed for flow cytometry. Dot plots and their respective bar graphs represent frequencies and absolute cell numbers of the specified subpopulations (Total CD8^+^ T cells, CD44^hi^CD8^+^ T cells and T_MEM_/T_EFF_ and T_VM_ cells among CD44^hi^CD8^+^ T cells) from control (left dot plots) or 12 + 18 mice (right dot plots) on **(A, C, E)** spleen or **(B, D, F)** dLN. Statistical analysis was performed with Student T test (lower **B–D** and lower **E**), or applying Welch **(A)** or Mann-Whitney (upper **B**, upper **E, F**) correction as appropriate. Values of **p<0.01, ****p<0.0001 were consider significant. ^####^ p<0.0001 represent the statistical difference of the indicated T_MEM_/T_EFF_ subpopulations. Ns, Not significant.

As shown in **Figure 2**, both the percentage and the absolute cell number of total CD8^+^ T cells in the spleen (**Figure 2A**) and dLNs (**Figure 2B**) did not change when comparing control and 12+18-treated mice. However, the treatment induced a significant increase in the proportion of CD8^+^CD44^hi^ T cells in both examined SLO (**Figures 2C, D**). Of note, it is important to mention that CD44 expression is a consensus marker for memory and activated T cells and it is highly expressed by both T_VM_ and T_MEM_/T_EFF_ cells (8, 34).

As previously reported, CD49d is the integrin subunit alpha 4 and is up-regulated after a strong signal through the TCR in T cells driven by a cognate antigen. This important marker allows one to distinguish T_VM_ cells from conventional effector memory T cells (3–5). Based on this receptor expression, we found that in the spleen of control mice, there are similar percentages of T_VM_ vs T_MEM_/T_EFF_ cells but after exposure to systemic IL-12+IL-18, T_MEM_/T_EFF_ cells expanded almost 3 times while the number of T_VM_ cells remained almost unchanged (**Figure 2E**). A similar pattern was observed in dLNs. Of note, T_VM_ are highly enriched in this tissue, representing approximately 80% of total CD8^+^CD44^hi^ cells in steady-state conditions (control mice) (**Figure 2F**). Overall, we observed that IL-12+IL-18 systemic expression preferentially favors the expansion of T_MEM_/T_EFF_ over T_VM_ cells in SLO.

**Figure 3:**
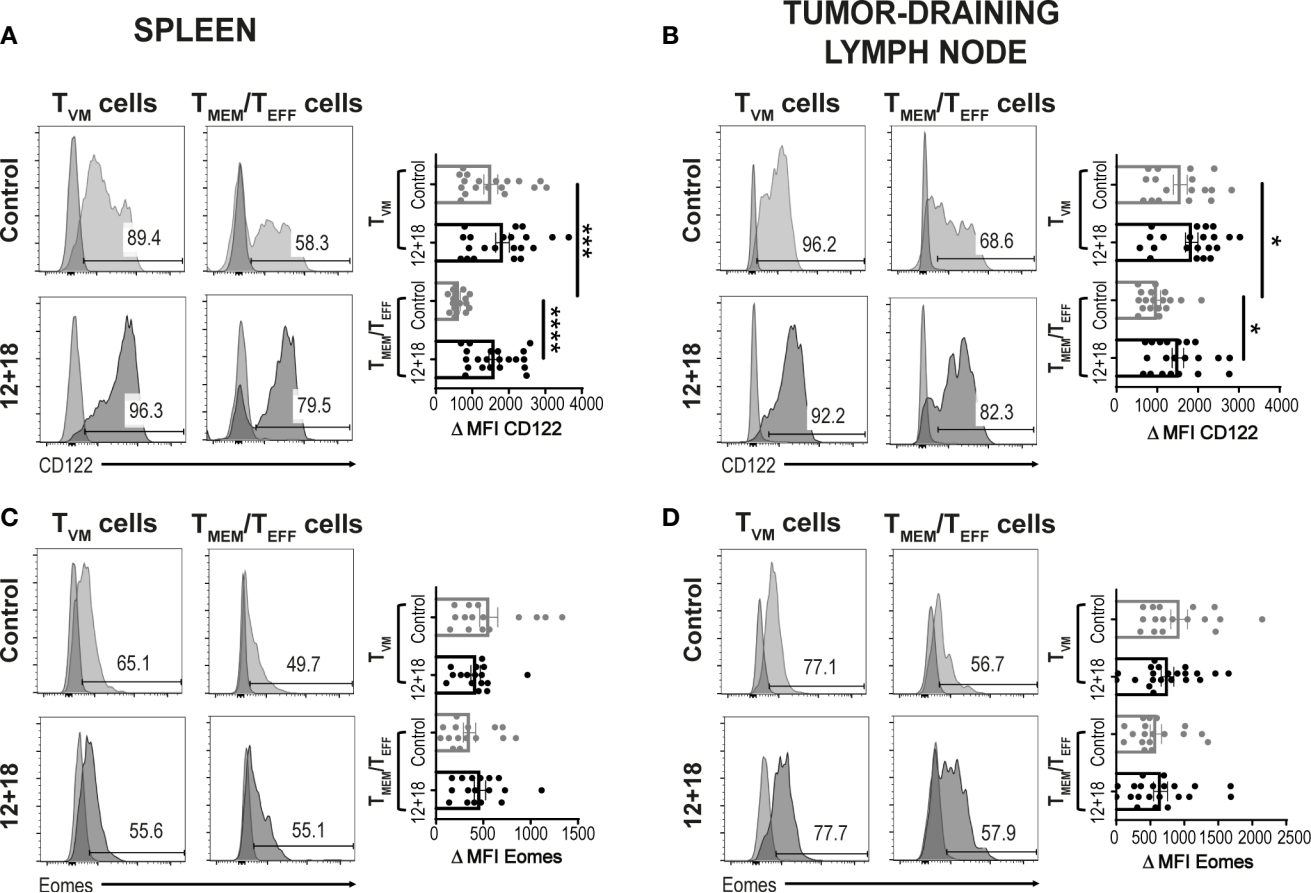
Effects of systemic co-expression of IL-12 plus IL-18 on T_VM_ and T_MEM_/T_EFF_ cells in SLO. B16-bearing C57BL/6 WT mice were hydrodynamically injected (HI) with IL-12 plus IL-18 cDNA (12 + 18) or empty cDNA as the control. After 7 days post-HI mice were euthanized, and spleen and tumor-draining lymph nodes (dLN) were harvested and processed for flow cytometry. Histograms and their respective bar graphs represent **(A, B)** CD122 or **(C, D)** Eomes frequencies and Δ medium fluorescence intensity (MFI) of T_VM_ (left histograms) and T_MEM_/T_EFF_ cells (right histograms) from control (upper histograms) or 12 + 18 mice (lower histograms) on **(A, C)** spleen or **(B, D)** dLN. Δ MFI was calculated as CD122 or Eomes MFI minus MFI from their respective isotype controls. Statistical analysis was performed with One-way ANOVA test **(B)** or Kruskall Wallis test **(A, C, D)**. Values of *p<0.05, ***p<0.001 ****p<0.0001 were consider significant.

Next, we evaluated if systemic IL-12+IL-18 treatment was able to alter the expression of the different consensus markers of T_VM_ and T_MEM_/T_EFF_ cells. Evaluation of CD122 expression demonstrated that this marker is already highly expressed on T_VM_ cells in the spleen (**Figure 3A**) and dLNs (**Figure 3B**) under steady-state conditions compared to conventional memory/effector CD8^+^ T cells, as previously reported (8, 34). However, after systemic expression of IL-12+IL-18, only T_MEM_/T_EFF_ cells up-regulate this marker to levels similar to that observed in T_VM_ cells. When we evaluated Eomes expression, we observed that both cell subsets expressed high levels of this transcription factor and expression levels were not altered by the *in vivo* treatment with the cytokines, both in the spleen and dLNs (**Figures 3C**, **3D**, respectively).

#### 3.2.2 Alterations in tumors

**Figure 4:**
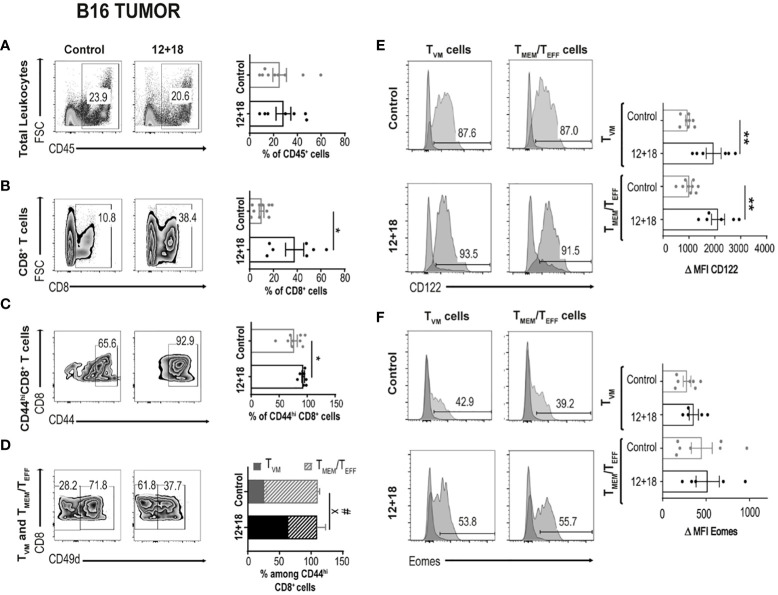
Effects of systemic co-expression of IL-12 plus IL-18 on leukocytes and CD8^+^ T lymphocytes in tumors. B16-bearing C57BL/6 WT mice were hydrodynamically injected (HI) with IL-12 plus IL-18 cDNA (12 + 18) or empty cDNA as the control. After 7 days post-HI mice were euthanized, and tumors were harvested and processed for flow cytometry. **(A–D)** Dot plots and their respective bar graphs represent the frequencies of the specified subpopulations (total leukocytes, total CD8^+^ T cells, CD44^hi^CD8^+^ T cells and T_MEM_/T_EFF_ and T_VM_ cells among CD44^hi^CD8^+^ T cells) from control (left dot plots) or 12 + 18 mice (right dot plots). **(E, F)** Histograms and their respective bar graphs represent CD122 (top) or Eomes (bottom) frequencies and Δ MFI on T_VM_ (left histograms) and T_MEM_/T_EFF_ cells (right histograms) from control (upper histograms) or 12+18 mice (lower histograms). Δ MFI was calculated as MFI of CD122 or Eomes minus MFI from their respective isotype control. Statistical analysis was performed with Student T test **(A)**, with Welch correction **(B–D)** or with a One-Way **(E)** or Brown Forsythe and Welch **(F)** ANOVA test as appropriate. Values of *p<0.05, **p<0.01, were consider significant. ^#^ p<0.05 represent the statistical difference of the indicated T_MEM_/T_EFF_ control vs 12 + 18 subpopulations while X p<0.05 represents the difference among control and 12 + 18 T_VM_ cells.

After evaluation of these parameters in SLO, we focused next on determining the frequency and phenotype of T_VM_ and T_MEM_/T_EFF_ cells infiltrating B16 tumors. We observed that the treatment did not alter the percentage of total CD45^+^ infiltrating leukocyte (TILs) (**Figure 4A**). However, the frequency of CD8^+^ T cells within TILs is significantly increased in mice that received IL-12+IL-18 *in vivo* (**Figure 4B**). We observed that most CD8^+^ T cells express high levels of CD44^hi^, and this percentage is even higher after IL-12+IL-18 treatment (**Figure 4C**). After the treatment with the cytokines, we expected to see an enrichment of T_MEM_/T_EFF_ cells similar to data obtained in SLO. However, in the tumor site, the frequency of T_VM_ cells increased from about 25% in control mice to more than 60% in 12 + 18-treated mice (**Figure 4D**). When we evaluated CD122 and Eomes expression in these cells, we obtained similar results to SLO, but in this case CD122 expression increased in both T_VM_ and T_MEM_/T_EFF_ cells after IL-12+IL-18 systemic expression (**Figure 4E**) while Eomes levels remained high and unaffected (**Figure 4F**).

### 3.3 Systemic IL-12 and IL-18 expression impacts the number and phenotype of T_VM_ and T_MEM_/T_EFF_ cells but not the antitumor capacity in IL-4KO and IFNAR KO mice

Previously, we have shown that expression of systemic IL-12 and IL-18 is able to induce IL-15 expression in sites where memory T cells usually reside. Moreover, we demonstrated how the frequency and phenotype of both T_VM_ and T_MEM_/T_EFF_ cells are affected both in SLO and tumors in a bystander mechanism after non-antigenic *in vivo* stimulation. Other than the mentioned cytokines, it has been reported that IL-4 and type I IFNs play a crucial role during development/maintenance and functional stages of T_VM_ cells in steady-state conditions (38, 39). However, their role after systemic Th1 inflammatory situations has not been completely addressed. To evaluate the impact of IL-4 and type I interferons, we examined the frequency and phenotype of T_VM_ and T_MEM_/T_EFF_ cells in SLO and the antitumor ability of systemic IL-12 and IL-18 expression in mice deficient in IL-4 or type I IFNs receptor (IFNAR). As shown in **Supplementary Figure 3**, we observed that the total CD8^+^ absolute T cell number were not affected after 12 + 18 treatment either in IL-4KO nor in IFNAR KO mice both in spleen (**Supplementary Figure 3A**) and dLNs (**Supplementary Figure 3B**).

As previously determined for WT mice, systemic expression of IL-12+IL-18 also increased the percentages of CD8^+^CD44^hi^ T cells in IL-4 KO. Surprisingly, in IFNAR KO mice, this increase is observed in spleen (**Supplementary Figure 3C**) but not in dLNs (**Supplementary Figure 3D**). An interesting result arose when comparing the frequency of T_VM_ vs T_MEM_/T_EFF_ cells between WT and the KO mice. In contrast to WT mice, we observed that the frequency of T_MEM_/T_EFF_ cells in IL-4 KO mice is similar in control and 12 + 18-treated mice. Unexpectedly, in IFNAR KO mice, T_MEM_/T_EFF_ cells were almost undetectable in control mice and even though the cells increased after IL-12+IL-18 treatment, they did not reach the proportion observed in WT mice (**Supplementary Figures 3E, F**).

These data demonstrate how the presence of these cytokines can regulate the normal proportions of T_VM_ and T_MEM_/T_EFF_ cells in SLO in steady-state and also during systemic Th1 inflammatory situations. In spite of the changes observed in the frequency of these cell populations, the antitumor capacity in both IL-4 and IFNAR KO mice was high and similar to WT mice (**Supplementary Figure 3G**).

### 3.4 Enrichment of T_VM_ cells by using OT-I mice

#### 3.4.1 The T_VM_ cells vs T_MEM_/T_EFF_ cells balance in SLO of WT and OT-I mice

**Figure 5:**
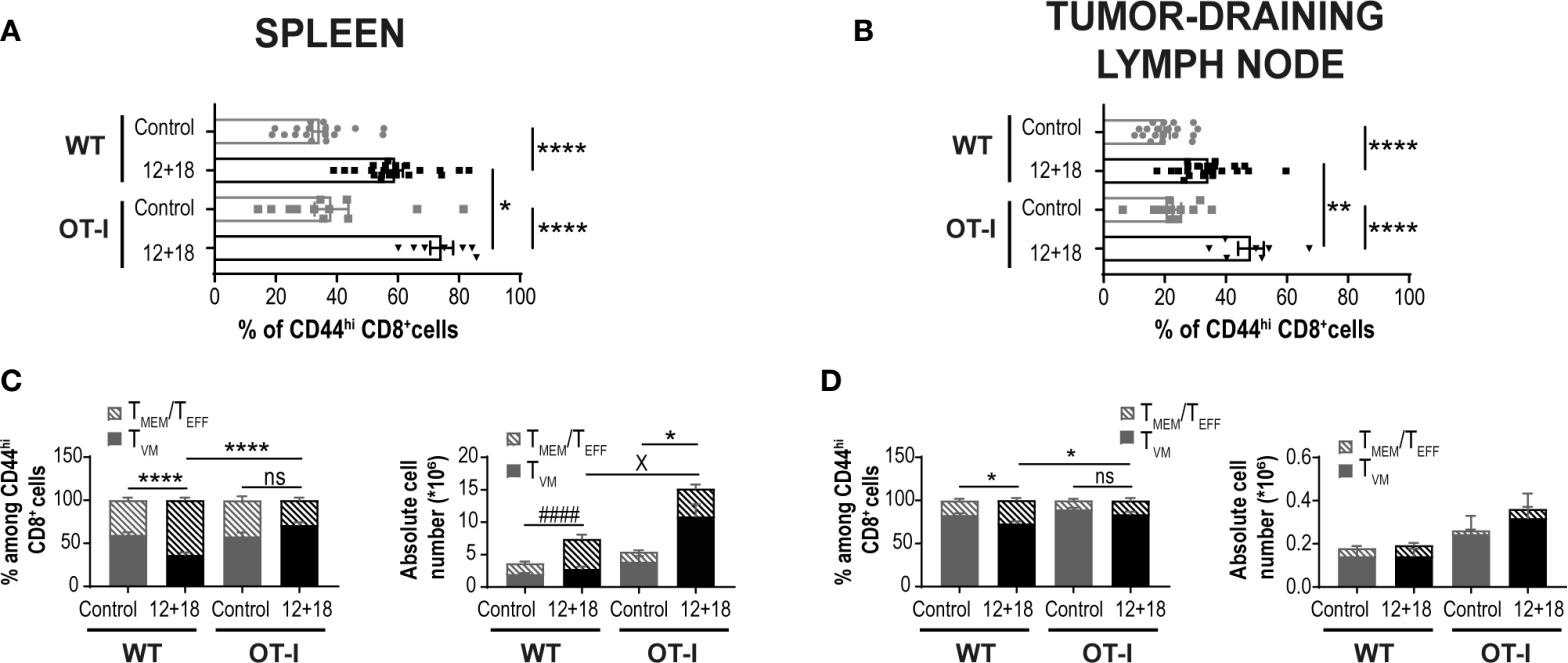
Effects of systemic co-expression of IL-12 plus IL-18 on CD8^+^ T lymphocytes in SLO from WT and OT-I mice. OVA^neg^ B16-bearing C57BL/6 WT and OT-I mice were hydrodynamically injected (HI) with IL-12 plus IL-18 cDNA (12 + 18) or empty cDNA as the control. After 7 days post-HI mice were euthanized, and spleen and dLN were harvested and processed for flow cytometry. **(A, B)** Bar graphs represent frequencies of CD44^hi^CD8^+^ T cells on **(A)** spleen or **(B)** dLN from control or 12 + 18 WT or OT-I mice. **(C, D)** Bar graphs show frequencies and absolute cell numbers of T_MEM_/T_EFF_ and T_VM_ cells among CD44^hi^CD8^+^ T cells from **(C)** spleen or **(D)** dLN from control or 12 + 18 WT or OT-I mice. Statistical analysis was performed with One-way ANOVA **(A-D)**. Values of *p<0.05, **p<0.01, ****p<0.0001 were consider significant. ^####^ p<0.0001 represents the statistical difference of the indicated T_MEM_/T_EFF_ control vs 12 + 18 subpopulations, while X p<0.01 represents the difference among the specified control and 12 + 18 T_VM_ cells, * indicates an statistical difference in both subpopulations of memory CD8^+^ T cells.

**Figure 6:**
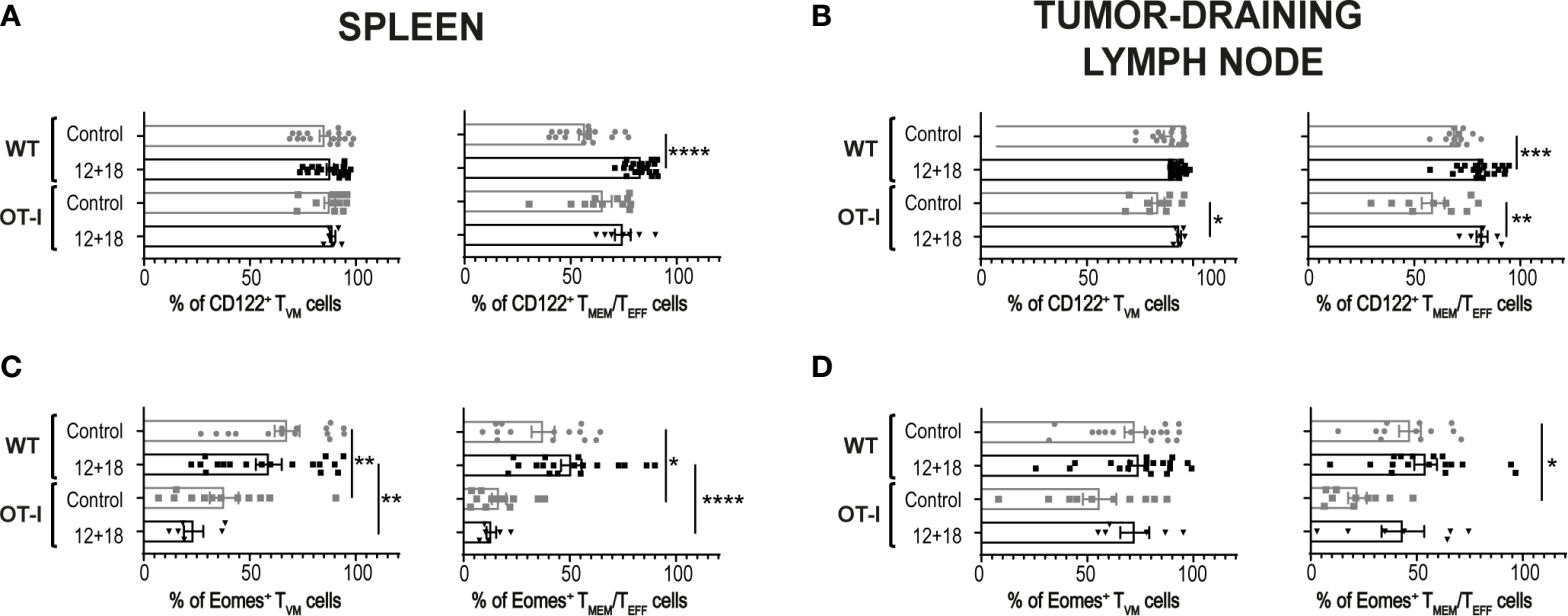
Effects of systemic co-expression of IL-12 plus IL-18 on T_VM_ and T_MEM_/T_EFF_ cells in SLO from WT or OT-I mice. OVA^neg^ B16-bearing C57BL/6 WT or OT-I mice were hydrodynamically injected (HI) with IL-12 plus IL-18 cDNA (12 + 18) or empty cDNA as the control. After 7 days post-HI mice were euthanized, and spleen and tumor-draining lymph nodes (dLN) were harvested and processed for flow cytometry. Bar graphs show the frequencies of **(A, B)** CD122^+^ or **(C, D)** Eomes^+^ T_VM_ or T_MEM_/T_EFF_ cells (left and right graphs respectively) on **(A, C)** spleen and **(B, D)** dLN from each experimental group. Statistical analysis was performed with One-Way ANOVA (A, D, left B, and left C) or Brown-Forsthy and Welch ANOVA test (right B and right C). Values of *p<0.05, **p<0.01, ***p<0.001, ****p<0.0001 were consider significant.

It has been proposed that the chances of a CD5^hi^CD8^+^ T cell that leaves the thymus to become a T_VM_ cell in the periphery depends upon the accessibility of certain niches in SLO where the appropriate conditions for their differentiation are present (8, 34, 40). Then, the chances for a specific T cell to become a T_VM_ cell not only depends upon the type of selection that it received in the thymus but also on the competition for niches in SLO. Based on these data, we decided to evaluate a scenario where the proportion of T_VM_ cells over T_MEM_/T_EFF_ is higher than in WT mice. We used non-RAG KO OT-I mice where most of the CD8^+^ T cells carry a TCR specific for OVA, a protein not expressed by B16 tumors. In these mice, T_VM_ and T_MEM_/T_EFF_ cells still co-exist but with a larger proportion of T_VM_ cells (OVA specific) than in WT mice (**Supplementary Figure 4**). Interestingly, when using this experimental model we observed that while the number of CD8^+^CD44^hi^ T cells is similar between WT and OT-I mice in the control groups, the frequency of these cell subsets increased in a larger proportion in OT-I compared to WT mice after IL-12+IL-18 *in vivo* stimulation in both SLO (**Figures 5A, B**). Furthermore, in OT-I mice the higher numbers of CD8^+^CD44^hi^ T cells after IL-12+IL-18 treatment is mainly due to an increase in absolute cell numbers of T_VM_ cells over T_MEM_/T_EFF_ cells in spleen (**Figure 5C**) and dLNs (**Figure 5D**). In addition, evaluation of CD122 and Eomes expression demonstrated similar results when comparing WT and OT-I mice with an up-regulation of CD122 in T_MEM_/T_EFF_ cells after the 12 + 18 treatment, especially in dLNs (**Figures 6A, B**). Surprisingly, Eomes expression in both T_VM_ and T_MEM_/T_EFF_ cells is lower in OT-I than in WT mice, especially in spleen (**Figure 6C**) and is not affected by the treatment (**Figures 6C, D**).

When we performed a non-supervised flow cytometry analysis (Trimap) of these cell subsets, we observed similar results to the supervised data (shown in **Figures 5**, **6**). Furthermore, as shown in **Supplementary Figure 5**, T_MEM_/T_EFF_ cells predominate after IL-12+IL-18 treatment in WT mice while the opposite effect is seen in OT-I where T_VM_ cells are enriched after the cytokine treatment.

#### 3.4.2 High and similar antitumor capacity of OT-I and WT mice after systemic expression of IL-12 and IL-18

**Figure 7:**
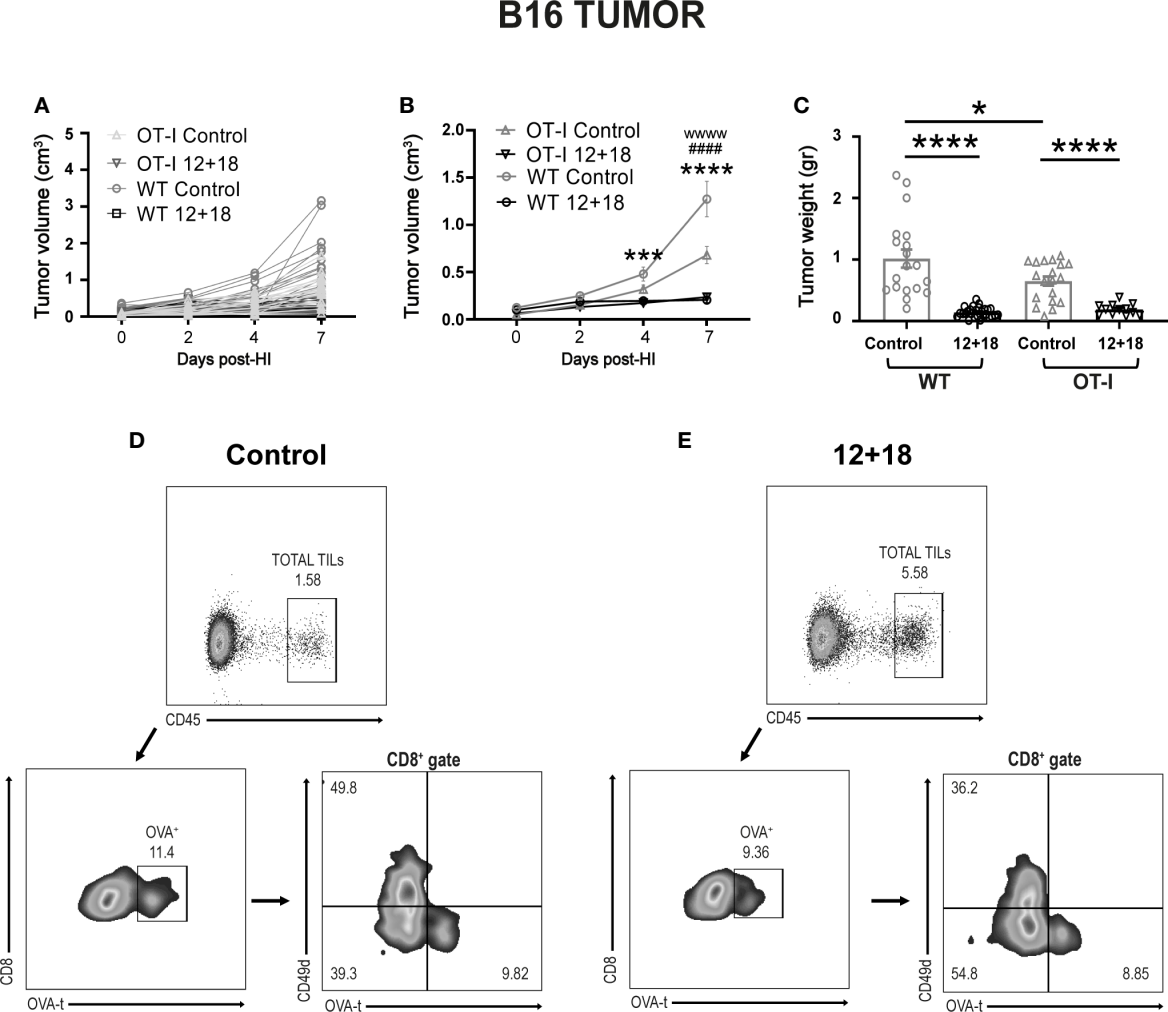
Effects of the systemic co-expression of IL-12 plus IL-18 on tumor growth on OT-I mice. OVA^neg^ B16- bearing C57BL/6 WT and OT-I mice were hydrodynamically injected (HI) with IL-12 plus IL-18 cDNA (12 + 18) or empty cDNA as the control. At the indicated times post-HI tumor size was measured using a caliper. After 7 days post-HI mice were euthanized, and tumors were harvested and weighed using an analytical scale. **(A, B)** B16 Tumor growth represented as tumor volume (in cm^3^) at specified days post-HI is shown **(A)** for each mouse individually or **(B)** as an average (mean ± SEM) for each experimental group. **(C)** B16 tumor weight in grams is represented as mean ± SEM for each specified experimental group. **(D, E)** B16 tumors from **(D)** control or **(E)** 12 + 18-treated OT-I mice were processed and stained for flow cytometry using Zombie dye, anti-CD45, anti-CD8, anti-CD49d and OVA-tetramer. Dot plots represent the gating strategy used for analyzing the tumor infiltration of CD8+ OVA+ cells from one representative animal from each group. Statistical analysis was performed using a two-way ANOVA with Sidak’s multiple comparison test on **(B)** or a One-way ANOVA test **(C)**. Values of *p<0.05, ***p<0.001, ****p<0.0001 were consider significant. Values of ***, ****, wwww p<0.0001 y ^####^p<0.0001 on **(B)** represent the significant difference between control and 12 + 18 WT mice (*), control and 12 + 18 OT-I mice (w) or between WT and OT-I control mice (^#^) respectively.

When we compared the antitumor ability against B16 in OT-I and WT mice we observed that tumor growth is significantly lower in control OT-I than WT mice in the days post-treatment (**Figures 7A, B**). This effect is also confirmed by smaller tumors in OT-I control mice at day 7 post-treatment (**Figure 7C**). Although we observed a certain amount of CD4^+^ T cells in the non-RAG KO OT-I mice (**Supplementary Figures 4, 7**), we have not evaluated the proportion of conventional vs regulatory CD4+ T cells (Treg) cells and anti-tumor activity in control mice. However, the antitumor capacity is similar and highly efficient after IL-12+IL-18 systemic expression in both strains of mice (**Figures 7A–C**). Interestingly, we confirmed that a proportion of CD45^+^ cells infiltrating the B16 tumors from OT-I are OVAt^+^ cells (~9-10% of total CD8^+^ cells) (**Figure 7D**). Moreover, IL-12+IL-18 stimulation increased the frequency of polyclonal T_VM_ cells (although OVAt^+^ cells are in similar percentage compared to control mice) (**Figure 7E**). Importantly, OVAt^+^ cells are CD49d^lo^ meaning that, at least in case of dual TCRs, they don´t carry a TCR specific for tumor antigens or are activated by other antigens.

**Figure 8:**
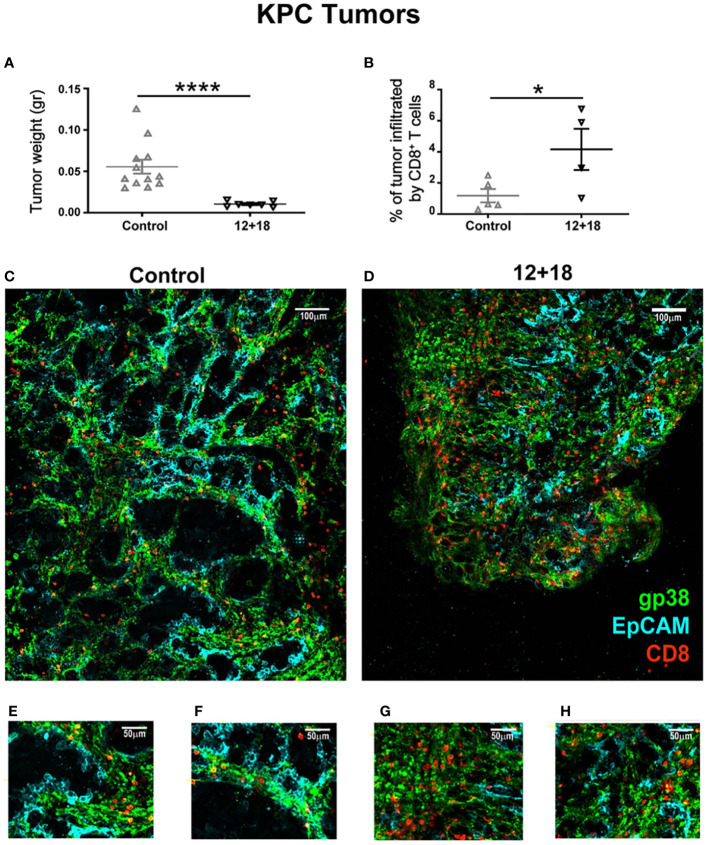
Effects of IL-12 plus IL-18 systemic expression on CD8^+^ T cell that infiltrate KPC tumors on OT-I mice. OVA^neg^ KPC- bearing C57BL/6 OT-I mice were hydrodynamically injected (HI) with IL-12 plus IL-18 cDNA (12 + 18) or empty cDNA as the control. After 7 days post-HI mice were euthanized, and tumors were harvested, weighed, fixed and included in a 5% agarose solution. Slides were made of 200µm using a Leica VT 1000S Vibratome and were stained with anti-CD8 (red), anti-gp38 (green) for stroma detection, anti-EpCAM (blue) for detecting epithelial (tumoral) cells. Images were taken with a DM500B upright microscope equipped with a SP5 confocal head (Leica). **(A)** KPC tumor weight at day 7 post-IH is shown from control or 12 + 18 OT-I mice. **(B)** Graph representing percentages of tumor area infiltrated by CD8^+^ T cells in each experimental group. Figures represent tumors from **(C, E, F)** control or **(D, G, H)** 12 + 18 mice, **(C, D)** reconstructing big tumor areas or **(E-H)** enlarging regions of interest with a 25x magnification. Statistical analysis was performed by Student T test **(A, B)**. Values of *p<0.05, ****p<0.0001 were consider significant.

We have previously demonstrated the potent antitumor capacity of IL-12+IL-18 cDNA (at higher doses) (27) or IL-12 cDNA systemic expression (25, 28) in B16 and other murine tumor models *in vivo* (3LL and EL4). In **Figure 4** we show that there is a significant increment in total CD8^+^ T cell infiltration in B16 tumors after systemic expression of IL-12+IL-18. Based on these results, we evaluated CD8^+^ T cell infiltration in tumors that have poor leukocyte infiltration due to the development of mechanisms of T cell exclusion. We have chosen the OVA^neg^ KPC cell line (pancreatic ductal adenocarcinoma) model because it demonstrates an inflammatory infiltrate with a scarcity of effector T cells (41). We asked if in this “cold” tumor model, systemic IL-12+IL-18 treatment was able to increase the capacity of CD8^+^ T cells to infiltrate the tumors. Moreover, this cell line, by not undergoing the epithelial-mesenchymal transition process, is able to recapitulate the biology of human cancers in mice as characterized by islets of tumor cells surrounded by stroma (**Supplementary Figure 6**). This type of structure allows us to differentiate not only the location, but also the behavior and migratory pattern of the cells that infiltrate the tumors. We first evaluated tumor sizes in KPC-bearing OT-I mice and similar to the B16 tumor model, KPC tumors were significantly smaller in 12 + 18-treated mice than in control mice (**Figure 8A**).

The results shown in **Figure 5** (OT-I mice) demonstrate that when T_VM_ cells predominate over T_MEM_/T_EFF_ cells, a higher antitumor capacity is observed. By using an OVA tetramer (OVAt), we evaluated the exclusive role of T_VM_ cells in KPC-bearing mice by selecting OT-I animals that carry 99-100% CD8^+^OVAt^+^ T cells where CD8^+^ T cells carry a TCR non-specific for tumor antigens (**Supplementary Figure 7**).

In this experimental setting, we observed that tumors from 12 + 18-treated mice were highly infiltrated by T_VM_ cells as compared to control mice (quantification in **Figure 8B** and representative images in **Figures 8C, D**). Magnification images (**Figures 8E–H**) show that in both groups of mice, T_VM_ cells are quite restricted to the stromal areas. However, in 12 + 18-treated mice, T_VM_ cells were able to infiltrate more efficiently into the tumor areas compared to tumors from control mice (**Figures 8G, H**).

**Figure 9:**
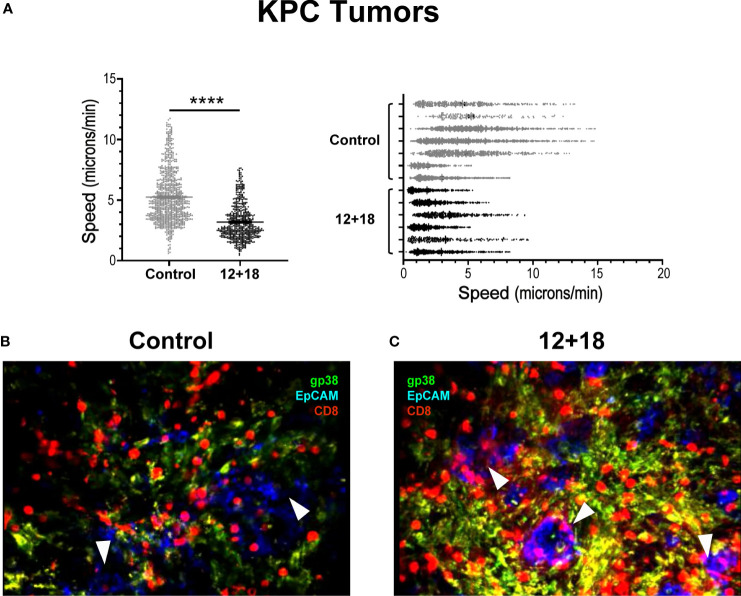
Effects of IL-12 plus IL-18 systemic expression on the behavior of tumor infiltrating CD8^+^ T cells from OT-I mice. OVA^neg^ KPC- bearing C57BL/6 OT-I mice were hydrodynamically injected (HI) with IL-12 plus IL-18 cDNA (12 + 18) or empty cDNA as controls. After 7 days post-HI mice were euthanized, and tumors were harvested. Tumors were immediately included in a 5% agarose solution. Slides were made of 200µm using a Leica VT 1000S Vibratome and were stained with anti-CD8 (red), anti-gp38 (green) for stroma detection, anti-EpCAM (blue) for detecting epithelial (tumoral) cells. Time lapse images were taken every 30 seconds for 20 minutes with a DM500B upright microscope equipped with a SP5 confocal head (Leica) and a 37°C thermostated chamber. **(A)** Graph shows the speed mean (microns/min) of each individual CD8^+^ T cell across all the mice (left graph) or from each mouse individually (right graph) with the average speed corresponding to each experimental group (mean ± SEM). **(B, C)** Images selected from two representative movies obtained from **(B)** control or **(C)** 12 + 18 KPC tumors show the infiltrate and distribution of CD8^+^ T cells. Statistical analysis was performed by Student T test with Mann-Whitney correction (left **C**). Values of ****p<0.0001 were consider significant.

To analyze the behavior of the lymphocytes inside the tumor, we performed *ex vivo* real-time imaging experiments in which we observed that T_VM_ cells from tumors of control animals showed a high mobility (**Supplementary Video 1**) as compared to those present in tumors from 12 + 18-treated mice, where the cells were visualized as considerably more static (**Supplementary Video 2**) (statistical analysis in **Figure 9A**). In selected images obtained from the videos, it can be clearly observed that T_VM_ cells in control animals are mostly restricted to stromal areas (green) (**Figure 9B**), while those of 12 + 18 animals are in close contact with the tumor islets (blue) (**Figure 9C**).

### 3.5 The antitumor effect of systemic IL-12+IL-18 is mainly mediated by IFNγ production

Due to the results obtained from videos 1 and 2, it seems that high infiltration into tumor islets along with low motility of T_VM_ cells indicate that cell-cell interactions may be an important part of the antitumor mechanism after systemic IL-12+IL-18 expression. The main candidate for this cell-cell interaction that has been previously described is NKG2D, a cell surface protein that is expressed in T_VM_ cells. However, the fact that most of the cell lines that were used in our experiment do not express NKG2D ligands made this hypothesis unlikely (26). In contrast, IFNγ production is synergistically produced in cells that simultaneously express IL-12R and IL-18R as in the case of T_VM_ cells (3, 12–14). Moreover, IFNγ has been demonstrated to induce a strong anti-angiogenic effect on tumor models (24). Based on these findings, we hypothesized that IFNγ could be playing a main role in this system as an antitumor effector. Interestingly, T_VM_ cells produce large amount of IFNγ in response to IL-12+IL-18 as previously demonstrated in infectious disease murine models (3, 12–14).

**Figure 10:**
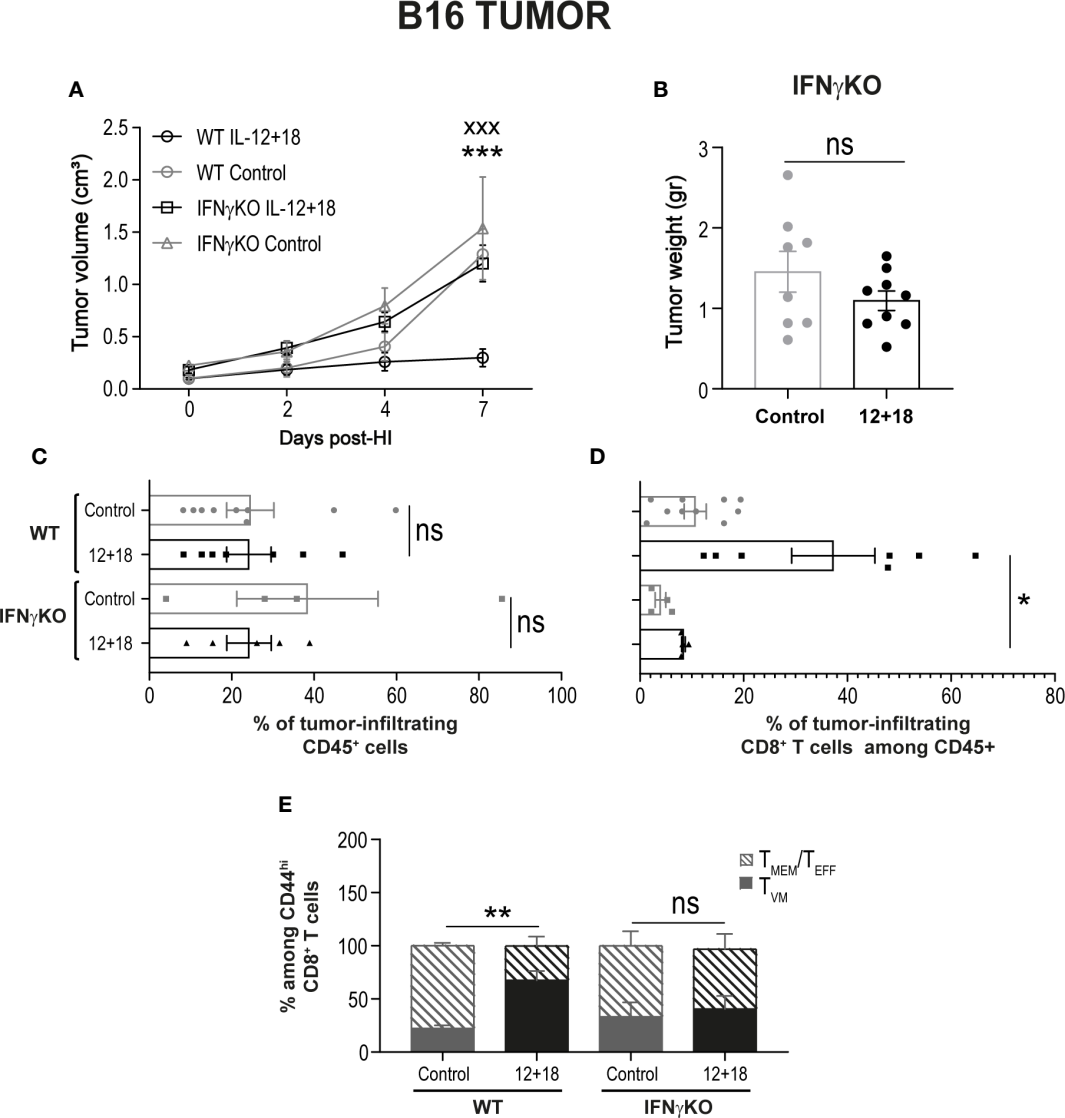
Effects of the systemic co-expression of IL-12 plus IL-18 on tumor growth on animals deficient on IFNγ. B16- bearing C57BL/6 WT and IFNγKO mice were hydrodynamically injected (HI) with IL-12 plus IL-18 cDNA (12 + 18) or empty cDNA as the control. At the indicated times post-HI tumor size was measured using a caliper. After 7 days post-HI mice were euthanized, and tumors were harvested, weighed using an analytical scale and processed for flow cytometry using a Zombie dye, anti-CD45, CD8, CD44, and CD49d. **(A)** B16 Tumor growth represented as tumor volume (in cm^3^) at specified days post-HI is shown as an average (mean ± SEM) for each experimental group. **(B)** B16 tumor weight in grams is represented as mean ± SEM for each specified experimental group. **(C–E)** Bar graphs represent the frequencies of **(C)** CD45^+^ infiltrating leukocytes, **(D)** CD8^+^ cells, or **(E)** T_VM_ and T_MEM_/T_EFF_ frequencies among memory CD44^hi^CD8^+^ cells. Statistical analysis was performed using a two-way ANOVA multiple comparison test with Sidak correction on **(A)**, a Student T test **(B)**, One-way ANOVA Test **(C, E)** or Brown-Forsythe and Welch ANOVA test **(D)** as appropriate. Values of *p<0.05, **p<0.01, ***p<0.001, and xxx p<0.001 were consider significant and represent the difference between control and 12 + 18 WT mice (*), or between 12 + 18 WT and IFNγKO mice (x), respectively. Ns, Not significant.

To test this hypothesis, we first evaluated tumor growth and size after systemic expression of IL-12+IL-18 in B16-bearing WT or IFNγKO mice (GKO). As shown in **Figure 10A**, the potent antitumor capacity of IL-12+IL-18 is completely abolished in mice that lack IFNγ. This effect is also visualized by similar tumor size when comparing control and 12 + 18-treated GKO mice (**Figure 10B**). When we evaluated the composition of TILs, we observed that even though the total leukocyte numbers were similar between WT and GKO mice in steady-state and Th1 inflammatory conditions (**Figure 10C**), the total number of infiltrating CD8^+^ T cells is highly diminished in GKO mice (**Figure 10D**). More interestingly, the large proportion of T_VM_ cells observed in WT mice after IL-12+IL-18 treatment is not seen in GKO mice as they exhibited similar values to WT control mice (**Figure 10E**).

## 4 Discussion

In the field of cancer immunology, the role of CD8^+^ T cells has been extensively addressed over the years. Solid tumors are usually infiltrated by different types of leukocytes and during the last 20-30 years, accumulating evidence has suggested that there is a positive correlation between tumor CD8^+^ T cells infiltration and a better prognosis for cancer patients. Moreover, this correlation is taken into account, both in the prognosis and in the choice of treatment for most cancers. For example, in colorectal cancer, Kirilovsky et al. have developed a prognostic score (‘Immunoscore’) in the classification system that takes into account the distribution of CD8^+^ cytotoxic T cells both in the tumor core and in the invasive margins that include tumor, lymph nodes and metastases (TNM) (22, 23).

Despite the clinical importance of the characterization of leukocytes in human and mouse tumors, there is a lack of information on the subtype of CD8^+^ T cells that infiltrate the tumors. For many years, it was assumed they are CD8^+^ T cells specific for Ags expressed by cancer cells. However, several reports suggest that CD8^+^ T cells with innate cell markers, not only infiltrate human and murine tumors, but also contribute to their eradication through mechanisms that are Ag-independent (11, 42, 43). Moreover, a recent review profoundly addresses the composition of tumor infiltrating CD8^+^ T cells and presents evidence demonstrating that a large fraction of human and murine tumor-infiltrating T cells are cancer unrelated and are designated as “bystanders T cells” (2). For example, Mognol et al. showed that, transferred activated “cancer-ignorant CD8^+^ cells” infiltrated murine tumors at levels comparable to those of cancer-specific CD8^+^ cells (44).

Comparative studies that simultaneously address T_MEM_ and T_VM_ cell differences and similarities in the cancer environment are rarely reported. Other than the work by Quinn et al. in humans (45), there is a complete absence of such reports in humans and the subject has only been approached by a few laboratories utilizing mouse models (4, 34, 46). However, it is important to note the work of Hussain et al. that presents a comprehensive comparative review of various aspects of the biology of both cell types (34). The appreciation of the role of T_VM_ cells as a first wave of immune protection, especially in infectious diseases, has become more obvious over the years. Moreover, the fact that T_VM_ cells can exhibit cytotoxicity more rapidly than naïve T cells, with similar kinetics and efficiencies of T_MEM_ cells (4, 45, 46), makes the investigation of T_VM_ cells very relevant in the cancer field.

Even though T_VM_ cells carry a functional TCR, these cells exhibit particularly high responsiveness to cytokines including IL-12, IL-18 and IL-15 as compared to specific Ag stimulation. Moreover, both Ag-inexperienced (T_VM_) and Ag-experienced (T_MEM_ and T_EFF_) cells proliferate in response to IL-15 and upon stimulation by IL-12 and IL-18 can elicit TCR independent proliferation, cytokine production and bystander cytotoxicity (4, 9, 34, 47, 48).

Based on these reports and the significant lack of information on the competitive role of T_VM_ vs T_MEM_/T_EFF_ cells in cancer, we aimed to investigate the prevalence of T_VM_ vs T_MEM_/T_EFF_ cells in SLO from mice bearing early established tumors, both in steady-state conditions and after systemic expression of IL-12, IL-18 and IL-15.

In our models, mice bear either B16 or KPC tumors for no longer than 8 to 10 days before cytokine induction. Moreover, the rapid antitumor response observed as early as 4 days (and up to 7 days) post-IL-12+IL-18 treatment leads us to speculate that the composition of the CD44^hi^CD8^+^ T cell subset is a mix of T_VM_ cells (CD49d^lo^) and Ag-specific CD8^+^ T effector cells and possibly early T_MEM_ cells (CD49d^hi^). Thus, this provides us with a model where these memory/activated cells co-exist.

As previously reported, the constitutive high expression of IL-12R, IL-18R and CD122 situates T_VM_ cells in an advantagous position to rapidly respond to these cytokines (10, 49). Unexpectedly, our data demonstrate that systemic induction of IL-12+IL-18 (and IL-15) led to an enrichment of T_MEM_/T_EFF_ cells with high expression of CD122 in SLO while T_VM_ cells remained at similar numbers and phenotype as compared to control mice. Even though we predicted the opposite results would be found, our data are similar to what is reported by Akue et al. The authors state that the frequency of pre-existing T_VM_ cells is stable in both steady-state conditions and after a greatly expanded Ag-driven memory CD8^+^ T cell immune response (47).

Another explanation for the prevalence of T_MEM_ cells after systemic IL-12+IL-18 expression could arise from the fact that T_VM_ cells in WT mice are a polyclonal population of memory-like CD8^+^ T cells. As such, after contacting their cognate Ag, these cells could convert to T_EFF_ and T_MEM_ cells as reported during influenza and listeria infections (4, 15). In our tumor models, the prevalence of T_MEM_ cells after Th1 systemic conditions could be the result of conversion of tumor-specific T naïve (T_N_) cells plus tumor-specific T_VM_ cells to a T_EFF_/T_MEM_ phenotype. To test this hypothesis, we performed similar experiments utilizing non-RAG OT-I mice. In this model system (**Supplementary Figure 4**), we increased the number of T_VM_ cells (non-specific for tumor Ags) while a remnant number of polyclonal T_MEM_/T_EFF_ are still present. Interestingly under these conditions, after IL-12+IL-18 expression we observed a significantly larger number of CD8^+^CD44^hi^ T cells in OT-I mice than what was observed in WT mice. This population was mainly composed of T_VM_ rather than T_MEM_/T_EFF_ cells, especially in the spleen. An interesting finding was that after IL-12+IL-18 treatment, the levels of Eomes do not change compared to the levels in control mice both in T_VM_ and in T_MEM_/T_EFF_ cells. More surprisingly, in OT-I mice, Eomes levels are significantly lower in T_VM_ and T_MEM_/T_EFF_ from spleen but not in LNs as compared to WT mice. This is an unexpected finding since it has been reported that Eomes is able to bind to the *il2rb* promoter leading to increases in CD122 expression, thus driving increased T_VM_ cell sensitivity to IL-15 (6). This result led us to assume that up-regulation of CD122 in T_MEM_/T_EFF_ cells after the cytokine treatment correlated with higher Eomes expression in this population. However, other stimuli could be responsible for CD122 up-regulation in T_MEM_ cells as it is reported that TCR signaling augments IL-2Rβ expression *via* both transcriptional and post-transcriptional regulation (50).

Interestingly, despite the higher proportion of T_MEM_/T_EFF_ than T_VM_ cells in spleen and tumor-draining lymph nodes after IL-12+IL-18 expression, this is not what is observed on CD8^+^CD44^hi^ T cells infiltrating the tumors. These cells show a predominant presence of T_VM_ cells after cytokine expression. We could not determine if this effect is due to a preferential migration of T_VM_ cells to tumors under Th1 conditions or if the permanence/survival within tumors is favored by T_VM_ cells over T_MEM_/T_EFF,_ similar to what has been reported by Miller et al. with T_N_ cells. Those authors demonstrated that when they co-transferred polyclonal CD8 T_N_ cells and polyclonal CD8 T_VM_ cells into TRAMP bearing mice, they found that donor T_VM_ cells constituted a substantial fraction of the tumor-infiltrating CD8^+^ T cells 4 months later (40).

The rapid antitumor immune response observed after systemic expression of IL-12+IL-18 points out to innate effector mechanisms rather than a TCR-mediated effect. The Ag-independent mechanisms achieved by bystander CD8^+^ T cells might rely on NKG2D-ligands recognition (10, 11), granzyme/perforin release (15, 16) and high IFNγ production (3, 12–14). The fact that B16 cells do not express ligands for NKG2D (51) may indicate that this pathway is not involved in the control of B16 tumor growth. Instead, rapid production of IFNγ, driven by IL-12+IL-18 could be responsible for the antitumor results observed in the experimental settings described here. Interestingly, we found that either IL-12 alone or IL-12+IL-18 systemic expression generated almost a complete disappearance of NK cells at 7-10 days post-treatment (25, 28) indicating that expression of IFNγ can largely arise from T cells. In this context we speculated that IFNγ produced by CD8^+^ T cells could be responsible for this early antitumor effect, especially since previous reports have demonstrated that IFNγ participates in different stages of tumor growth control (24, 52, 53). In our laboratory, we have previously documented that high expression of IFNγ is systemically induced after IL-12 or IL-12+IL-18 expression (27). Furthermore, there is a significantly higher number of IFNγ^+^CD8^+^ T cells infiltrating B16 and EL-4 tumors after cytokine treatment compared to tumors from control mice (25). Data presented in this work demonstrates that in the absence of IFNγ, the strong antitumor outcome observed after IL-12+IL-18 systemic expression is completely lost and this effect correlates with a significantly lower presence of T_VM_ cells in the tumors of IFNγKO mice compared to WT mice.

As mentioned earlier, IFNγ is able to induce different antitumor mechanisms. For example, interferon-inducible protein 10 (IP-10)-induced by IFNγ can exert a potent anti-angiogenic effect (53). Also, it is reported that IFNγ produced within tumors suppresses VEGFR3 expression by acting directly on tumor vessel endothelial cells and on the tumor-infiltrating lymphocytes to indirectly alter endothelial cells’ VEGFR3 expression (24). Consistent with this effect, previous work from our laboratory has demonstrated a significant reduction in the number of blood vessels present in B16 and EL-4 tumors after systemic expression of IL-12 (25). Additionally, Dangaj et al., using murine and human experimental systems, demonstrated that the initial recruitment of T cells to tumors is driven by tumor-derived CCL5 along with IFNγ-inducible CXCR3 ligands secreted by myeloid cells present in the tumors (54). Interestingly, not only do we report here that IFNγ KO mice show a reduced CD8^+^ T cell infiltration but we have also demonstrated that IFNγ is produced mainly by infiltrating CD8^+^ T cells rather than by NK cells in B16 tumors after IL-12 systemic expression (25). Furthermore, our preliminary data indicates higher CCR5 expression on CD8^+^ T cells infiltrating B16 tumors from 12 + 18-treated mice, as compared to control mice (unpublished data).

To further demonstrate the antitumor role of T_VM_ cells, we developed a model utilizing the KPC tumor cell line that shows poor T cell infiltration. In this murine pancreatic tumor model, most T cells are excluded (41). Moreover, contrary to most murine tumor models, KPC tumors recapitulate the structure of human cancers, with the presence of tumor islets surrounded by stromal cells. By using OT-I mice, our study demonstrated that CD8^+^ T cells (99-100% OVA-specific CD8^+^ T cells) not only highly infiltrate KPC tumors but also preferentially localized in the tumor islet after IL-12+IL-18 expression, contrary to the control OT-I mice where most CD8^+^ cells are found in the stromal areas. Consistent with our finding, Kantari-Mimoun et al. have reported that activated CAR T cells triggered the up-regulation of ICAM-1 on tumor cells in an IFNγ-dependent pathway that enabled T cell entry into tumor islets (52).

Based on this evidence from our and other laboratories, we hypothesize that systemic IL-12+IL-18 is able to trigger, in turn, expression of IL-15 and IFNγ. This pro-inflammatory environment could establish the perfect scenario for tumor eradication by different Ag-independent mechanisms mediated preferentially by IFNγ-producing T_VM_ cells. We speculate that in these conditions, CCR5^+^T_VM_ cells can preferentially migrate or selectively persist in tumors and locally produce IFN ҄γ. The IFNγ could then stimulate an anti-angiogenic effect along with the induction of adhesion molecules that permit the entry of T cells into the tumor islet. Even though we have presented solid evidence that tumor growth control is largely associated to the prevalence of T_VM_ cells in the tumor environment, the limitation of this work is to exclusively point to T_VM_ as the cells responsible for this effect. Currently experiments in our laboratory are focused on developing a more appropriate experimental strategy to substantiate this hypothesis.

The control of tumor growth through Ag-independent pathways is a topic of growing interest, especially considering that several tumors lose the expression of MHC type I as an immune evasion mechanism (55, 56). This loss of expression makes the tumor less susceptible to Ag-specific lysis, but more susceptible to innate control mechanisms such as the one exerted by T_VM_ cells. Furthermore, as proposed by White et al. and Drobek et al., the T_VM_ pool does not stochastically originate from the naïve T cell repertoire; instead, it is derived from a subset of naïve T cells that present a high affinity for self-ligands (visualized in mice as T cells with high levels of CD5) (40, 57). In this context, it is interesting to hypothesize that the continuous low level of homeostatic TCR signaling could result as an important signal for their anti-tumor responses. Accordingly, OT-I T cells are reported to be relatively highly self-reactive (40, 57). Even though systemic IL-12+IL-18 is normally triggered in infectious diseases, their use as an antitumor treatment has been difficult to accomplish, largely due to toxicity. However, type I IFNs, IL-12, IL-15 and IL-18 are produced by activated antigen presenting cells and other cell types that are often present in the tumor microenvironment, thus suggesting that an innate immune response may be important in the host anti-tumor response (58, 59). Moreover, BATF3^+^ dendritic cells are predominant producers of IL-15. Curiously, tumor-residing BATF3^+^ dendritic cells have been shown to be required for T cell trafficking (60) that can ultimately participate in the recruitment of T_VM_ cells to tumors due to high avidity of T_VM_ cells for IL-15. Based on this evidence, it could be possible that alternative therapies, able to induce expression of these cytokine in the tumor microenvironment, may become quite promising for the direction of future therapeutic strategies.

## Data availability statement

The original contributions presented in the study are included in the article/**Supplementary Material**. Further inquiries can be directed to the corresponding author.

## Ethics statement

The animal study was reviewed and approved by Comité Institucional de Cuidado y Uso de Animales de Laboratorio of the Facultad de Ciencias Químicas of Universidad Nacional de Córdoba (CICUAL-FCQ).

## Author contributions

CS-F: Methodology, validation, formal analysis, investigation. MV: Methodology, validation, formal analysis. NB: Methodology, investigation. NL: Methodology, validation. QF: Methodology, validation. HY: Conceptualization, original draft, review and editing. LV: Methodology, validation, formal analysis. ED: Conceptualization, investigation, funding acquisition. MR-G: Conceptualization, methodology, investigation, supervision, project administration, funding acquisition, writing. All authors contributed to the article and approved the submitted version.

## Funding

This work was supported by Secretaría de Ciencia y Tecnología from Universidad Nacional de Córdoba (SECyT); Agencia Nacional de Promoción Científica y Tecnológica (ANPCyT); Fondo para la Investigación Científica y Tecnológica (FONCyT); Fundación para el Progreso de la Medicina and P-UE 22920160100116CO - CONICET. This work was supported in part by the Intramural Research Program of the Center for Cancer Research, National Cancer Institute (NCI), Cancer Innovation Laboratory (CIL) USA, under grant No. 1ZIABC009283-36. The views expressed in this article are those of the authors and do not necessarily reflect the official policy or position of the Department of Health and Human Services, nor does mention of trade names, commercial products, or organizations imply endorsement by the United States Government.

## Acknowledgments

The authors thank Diego Luti, Victoria Blanco, Cecilia Noriega, Dr. Soledad Miro, Sergio Oms and Dr. Ivanna Novotny, for animal care. Dr. Pilar Crespo and Dr. Paula Abadie for FACS technical support. Dr. Laura Gatica, Lic. Gabriela Furlan and Dr. Noelia Maldonado for cell culture support and Paula Icely for overall experimental technical assistance. We acknowledge the NIH Tetramer Core Facility for provision of the PE and APC-labeled SIINFEKL/Kb tetramers.

## Conflict of interest

The authors declare that the research was conducted in the absence of any commercial or financial relationships that could be construed as a potential conflict of interest.

## Publisher’s note

All claims expressed in this article are solely those of the authors and do not necessarily represent those of their affiliated organizations, or those of the publisher, the editors and the reviewers. Any product that may be evaluated in this article, or claim that may be made by its manufacturer, is not guaranteed or endorsed by the publisher.
