# Supplementary material for: Exploring the immunomodulatory role of virtual memory CD8+ T cells: Role of IFN gamma in tumor growth control
Source: Front Immunol. 2022 Oct 18;13:971001. doi: 10.3389/fimmu.2022.971001 (PMC9623162; doi:10.3389/fimmu.2022.971001)
Supplement: Supplementary file 3 [file DataSheet_1.docx]

Supplementary Material

## Supplementary Figures


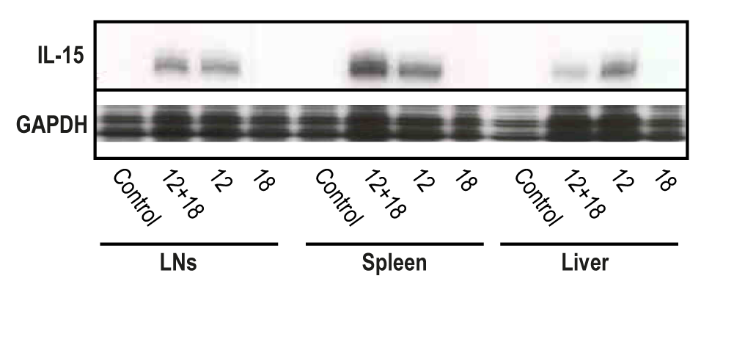


**Supplementary Figure 1.** **IL-15 mRNA expression on periphery in response to hydrodynamic injection of IL-12, IL-18 or a combination of both cytokines.** Tumor-free WT mice were hydrodynamically injected with control, IL-12 (12), IL-18 (18) or IL-12 plus IL-18 cDNAs (12+18) or injected with an empty cDNA as controls. After 72 h lymph nodes, spleen and liver were harvested and IL-15 mRNA expression was evaluated by RPA (BD Pharmingen multitemplate probe kit mck-1). Representative data from one of 3 independent experiments are shown.

**
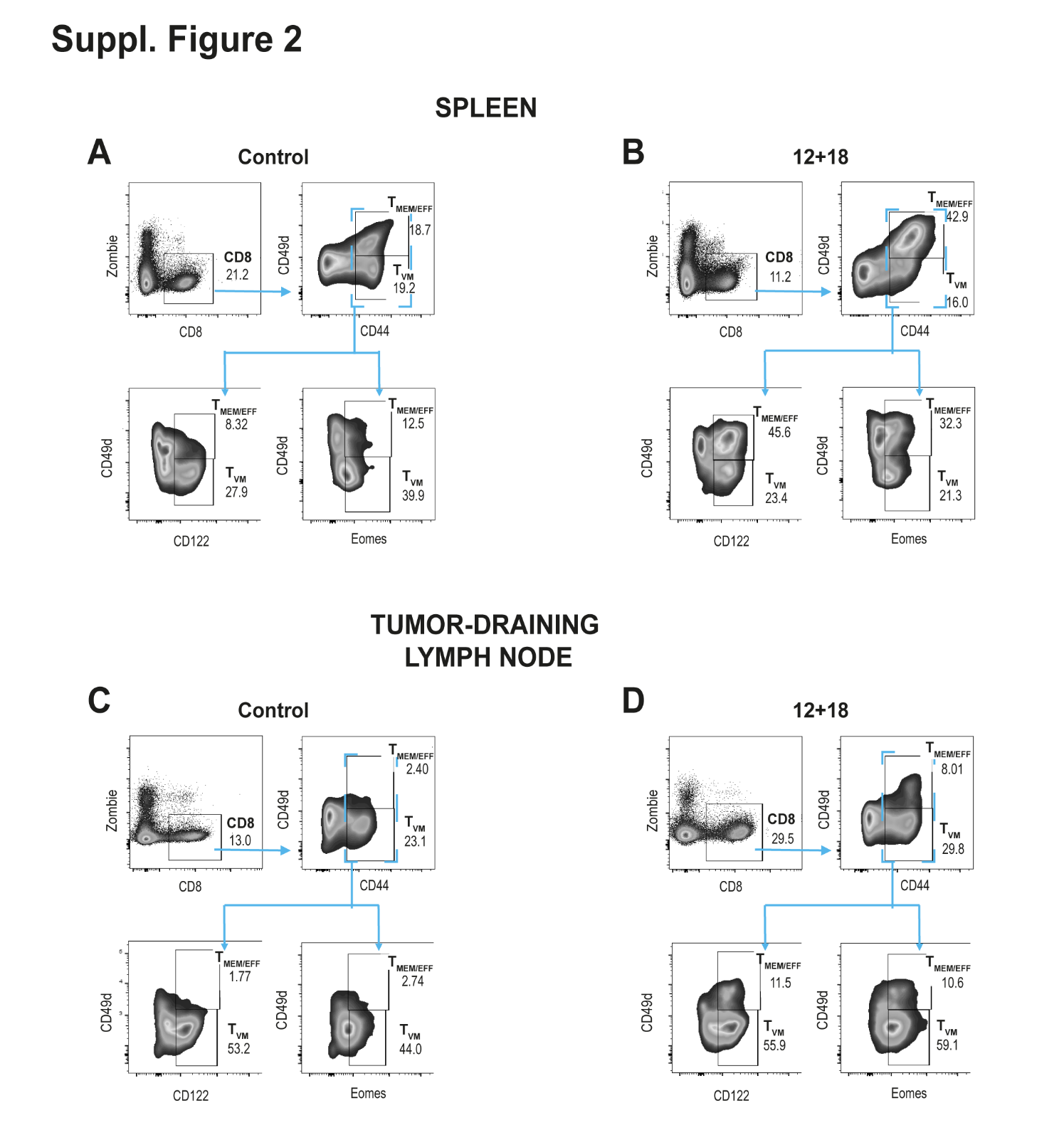
Supplementary Figure 2.** **Differential expression of CD44, CD122 and Eomes among T_MEM_/T_EFF_ versus T_VM_ cells in SLO in response to IL-12 plus IL-18.** B16-bearing C57BL/6 WT mice were hydrodynamically injected (HI) with IL-12 plus IL-18 cDNA (12+18) or empty cDNA as controls. After 7 days post-HI mice were euthanized, and spleen **(A, B)** and tumor-draining lymph nodes (dLN) **(C, D)** were harvested and processed for flow cytometry using Zombie dye and antibodies for detecting CD8, CD49d, CD44, CD122 and intracellular expression of Eomes. Dot plots represent the differential expression of the specified markers on T_MEM_/T_EFF_ versus T_VM_ cells among CD8^+^ T cells, considering T_MEM_/T_EFF_ cells as CD8^+^ CD44^hi^ CD49d^+^ and T_VM_ cells as CD8^+^ CD44^hi^ CD49d^neg^. Dot plots are shown for one representative animal from each experimental group: control **(A, C)** or 12+18 **(B, D)**.

**
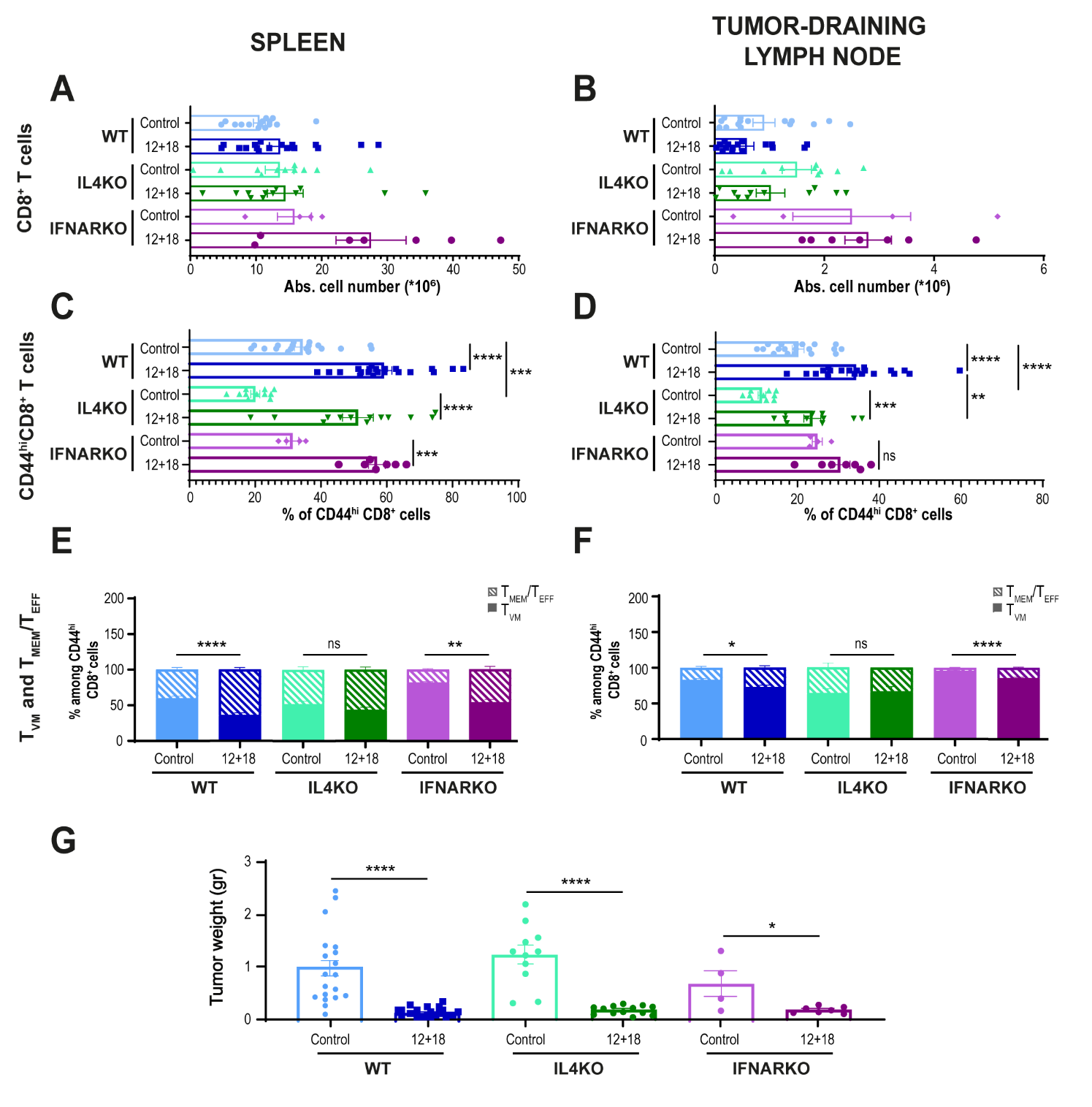
Supplementary Figure 3.** **Effects of systemic co-expression of IL-12 plus IL-18 on CD8^+^ T lymphocytes in SLO in the absence of IL-4 or type-I IFN receptors.** B16-bearing C57BL/6 WT, IL4KO and IFNARKO mice were hydrodynamically injected (HI) with IL-12 plus IL-18 cDNA (12+18) or empty cDNA as controls. After 7 days post-HI mice were euthanized, and spleen and tumor-draining lymph nodes (dLN) were harvested and processed for flow cytometry. **(A, B)** Bar graphs represent absolute cell numbers of CD8^+^ T cells or **(C, D)** frequencies of CD44^hi^CD8^+^ T cells on **(A, C)** spleen or **(B, D)** dLN from control or 12+18 WT (blue), IL4KO (green) or IFNARKO (violet) mice. **(E, F)** Bar graphs show frequencies of T_MEM_/T_EFF_ and T_VM_ cells among CD44^hi^CD8^+^ T cells from **(E)** spleen or **(F)** dLN from the indicated experimental group’s mice. **(G)** Tumor weight in grams at day 7 post-HI is shown for the different experimental groups. Statistical analysis was performed with One-way ANOVA (B, D, E and G) or Brown-Forsythe and Welch ANOVA test (A, C and F). Values of *p<0.05, **p<0.01, ***p<0.001, ****p<0.0001 were consider significant. Ns, not significant.


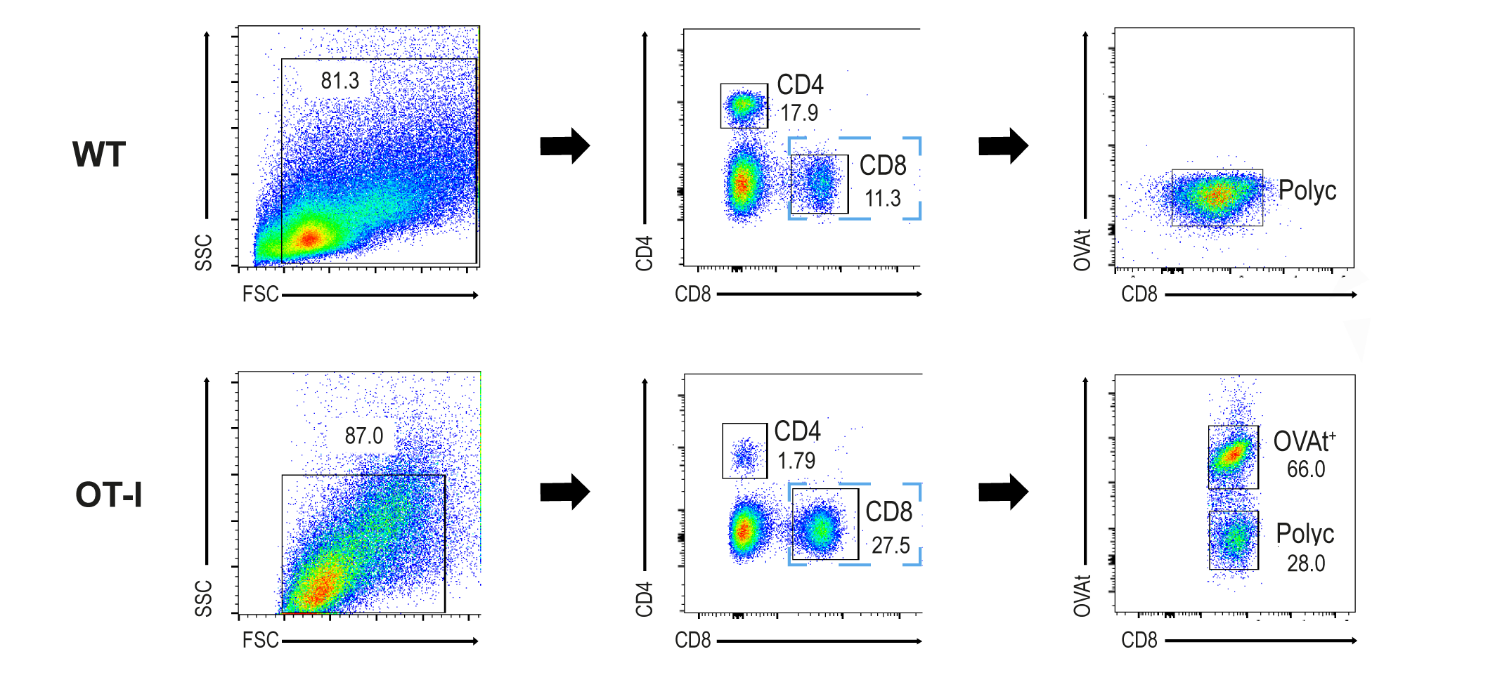


**Supplementary Figure 4.** **Heterogenic TCR-composition of CD8^+^ T cells from non-Rag2KO OT-I mice.** Non-Rag2KO OT-I and WT mice were submandibular bled and blood samples were first incubated with ACK lysis buffer for 10 minutes, washed with PBS and then stained for Flow Cytometry with Acqua Zombie dye, OVA-tetramer, and anti-CD8 and -CD4 antibodies. Cells were acquired on a BD LSR Fortessa and analysis was performed on FlowJo Software. Dot plots show the gating strategy used for this analysis. OT-I animals with around 70-80% of OVA-tetramer^+^ cells (OVAt^+^) and 20-30% polyclonal CD8^+^ T cells (Polyc) were used for experiments described in Figures 5, 6 and 7.


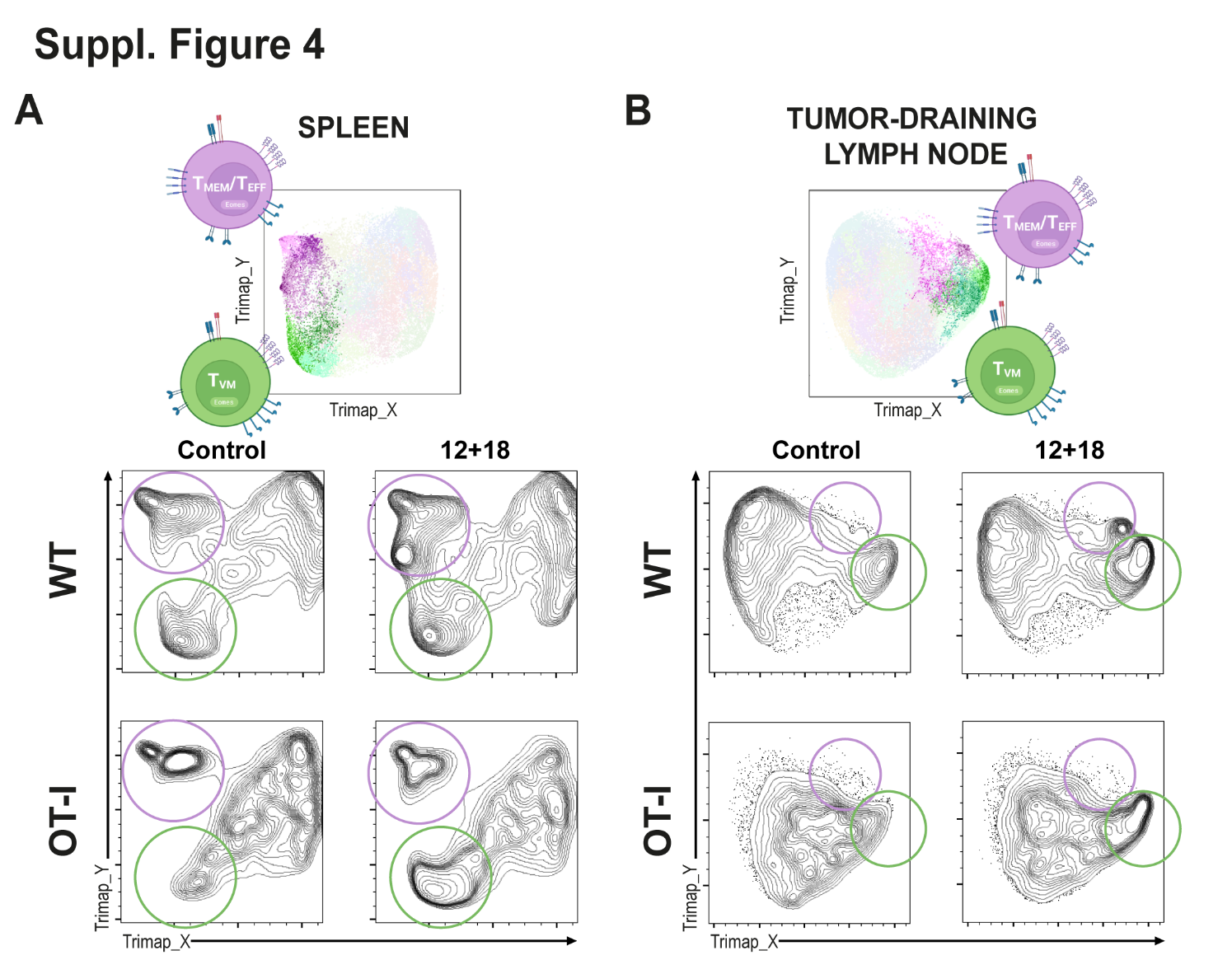


**Supplementary Figure 5.** **Non-supervised analysis of memory CD8^+^ T cells on SLO of control or 12+18 WT and OT-I mice.** OVA^neg^ B16-bearing C57BL/6 WT and OT-I mice were hydrodynamically injected (HI) with IL-12 plus IL-18 cDNA (12+18) or empty cDNA as controls. After 7 days post-HI mice were euthanized, and spleen and Tumor-draining lymph nodes (dLN) were harvested and processed for flow cytometry. Trimap and PhenoGraph dimensionality reduction analysis showing concatenated flow cytometry data of **(A)** spleen or **(B)** dLN from control- (left) or 12+18- (right graphs) treated WT and OT-I mice show the distribution of T_MEM_/T_EFF_ (purple) and T_VM_ cells (green) among total CD8^+^ T cells. T_MEM_/T_EFF_ cells were identified as CD44^hi^CD49d^+^ and T_VM_ as CD44^hi^CD49d^neg^. T_MEM_/T_EFF_ and T_VM_ cells illustration was created with BioRender.com.


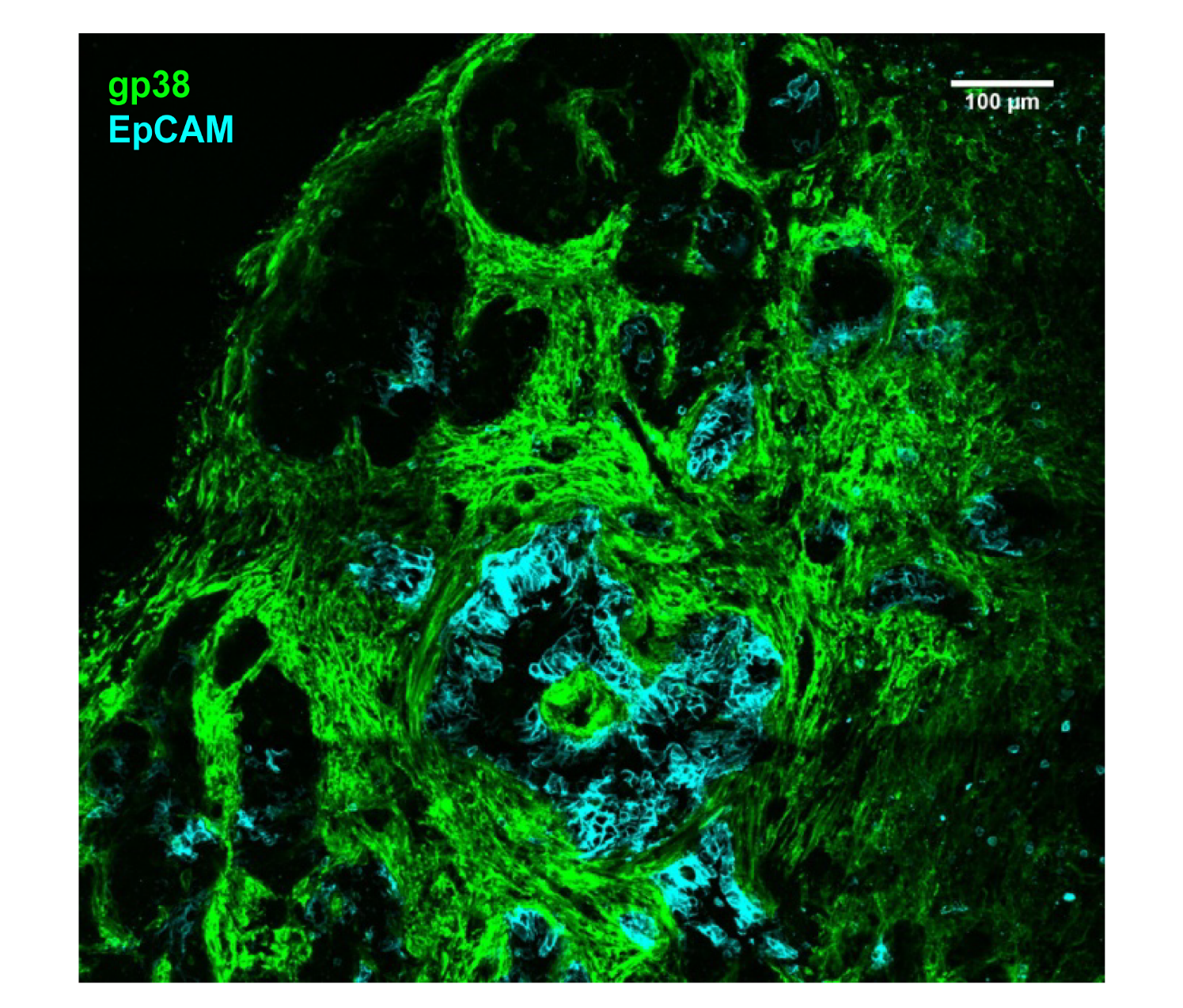


**Supplementary Figure 6.** **Microscopic structure of KPC tumors.** C57BL/6 OT-I mice were subcutaneously injected with 10x10^6^ KPC cells. Tumors were obtained after 14 days post-inoculation and were fixed and included in a 5% agarose solution. Slides were made of 200 μm using a Leica VT 1000S Vibratome and were stained with anti-gp38 (green) for stroma detection and anti-EpCAM (blue) for detecting epithelial (tumoral) cells. Images were taken with a DM500B upright microscope equipped with a SP5 confocal head (Leica). Image was processed with FIJI-ImageJ.


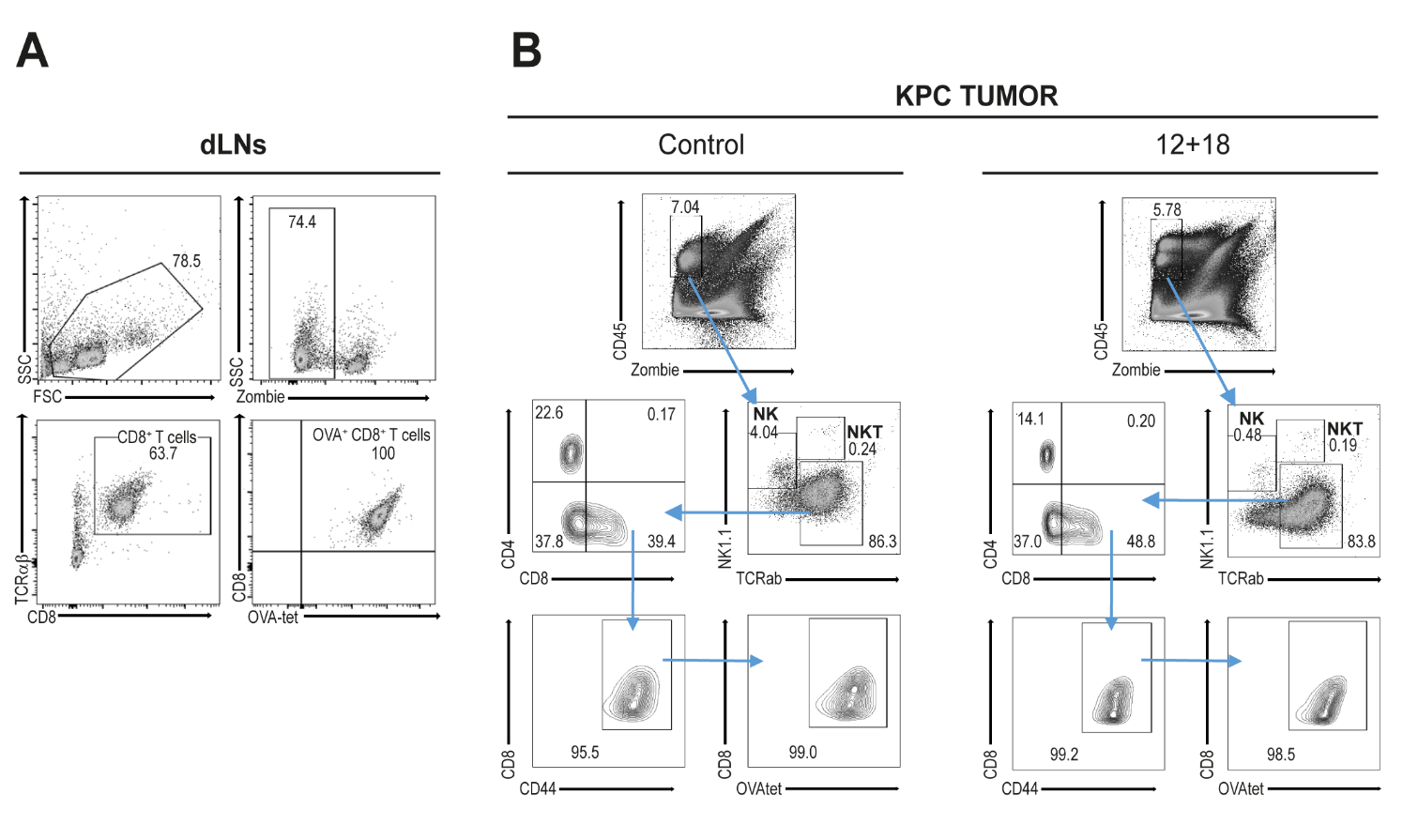


**Supplementary Figure 7.** **OT-I- KPC tumors and tumor-draining lymph nodes are enriched on OVA-specific CD8^+^ T cells.** OVA^neg^ KPC-bearing OT-I mice were hydrodynamically injected (HI) with IL-12 plus IL-18 cDNA (12+18) or empty cDNA as controls. After 7 days post-HI mice were euthanized, and tumor-draining lymph nodes (dLN) **(A)** or KPC tumors **(B)** were harvested and stained for Flow Cytometry with Zombie dye, OVA-tetramer, anti-TCRαβ, anti-CD45, anti-CD8, anti-CD4, anti-NK1.1, and anti-CD44 antibodies as indicated. Cells were acquired on a BD LSR Fortessa and analysis was performed on FlowJo Software. Gating strategy used for this analysis is shown. Animals with 99-100% of OVA-tetramer^+^ cells were used for experiments described in Figures 8 and 9 and Movies.

**Movies. Resident CD8^+^ T cells behavior on OVA^neg^ KPC tumors from control or 12+18 OT-I mice**. OVA^neg^ KPC-bearing C57BL/6 OT-I mice were hydrodynamically injected (HI) with IL-12 plus IL-18 cDNA (12+18) or empty cDNA as controls. After 7 days post-HI mice were euthanized, and tumors were harvested and immediately included in a 5% agarose solution. Slides were made of 200µm using a Leica VT 1000S Vibratome and were stained with anti-CD8 (red), anti-gp38 (stroma, green), anti-EpCAM (tumor islets, blue). Time-lapse images were taken every 30 seconds for 20 minutes with a DM500B upright microscope equipped with a SP5 confocal head (Leica) and a 37°C thermostatic chamber. The animation represents a three-dimensional (3D) reconstruction of a sequential *z* series performed on Imaris 7.4 (Bitplane AG). One representative video from each experimental group is shown.
